# Application of machine learning in measurement of ageing and geriatric diseases: a systematic review

**DOI:** 10.1186/s12877-023-04477-x

**Published:** 2023-12-12

**Authors:** Ayushi Das, Preeti Dhillon

**Affiliations:** 1 International Institute for Population Sciences, Deonar, Mumbai, 400088 India; 2 Department of Survey Research and Data Analytics, International Institute for Population Sciences, Deonar, Mumbai, 400088 India

**Keywords:** Machine learning, Geriatrics, Ageing

## Abstract

**Background:**

As the ageing population continues to grow in many countries, the prevalence of geriatric diseases is on the rise. In response, healthcare providers are exploring novel methods to enhance the quality of life for the elderly. Over the last decade, there has been a remarkable surge in the use of machine learning in geriatric diseases and care. Machine learning has emerged as a promising tool for the diagnosis, treatment, and management of these conditions. Hence, our study aims to find out the present state of research in geriatrics and the application of machine learning methods in this area.

**Methods:**

This systematic review followed Preferred Reporting Items for Systematic Reviews and Meta-Analyses (PRISMA) guidelines and focused on healthy ageing in individuals aged 45 and above, with a specific emphasis on the diseases that commonly occur during this process. The study mainly focused on three areas, that are machine learning, the geriatric population, and diseases. Peer-reviewed articles were searched in the PubMed and Scopus databases with inclusion criteria of population above 45 years, must have used machine learning methods, and availability of full text. To assess the quality of the studies, Joanna Briggs Institute's (JBI) critical appraisal tool was used.

**Results:**

A total of 70 papers were selected from the 120 identified papers after going through title screening, abstract screening, and reference search. Limited research is available on predicting biological or brain age using deep learning and different supervised machine learning methods. Neurodegenerative disorders were found to be the most researched disease, in which Alzheimer’s disease was focused the most. Among non-communicable diseases, diabetes mellitus, hypertension, cancer, kidney diseases, and cardiovascular diseases were included, and other rare diseases like oral health-related diseases and bone diseases were also explored in some papers. In terms of the application of machine learning, risk prediction was the most common approach. Half of the studies have used supervised machine learning algorithms, among which logistic regression, random forest, XG Boost were frequently used methods. These machine learning methods were applied to a variety of datasets including population-based surveys, hospital records, and digitally traced data.

**Conclusion:**

The review identified a wide range of studies that employed machine learning algorithms to analyse various diseases and datasets. While the application of machine learning in geriatrics and care has been well-explored, there is still room for future development, particularly in validating models across diverse populations and utilizing personalized digital datasets for customized patient-centric care in older populations. Further, we suggest a scope of Machine Learning in generating comparable ageing indices such as successful ageing index.

**Supplementary Information:**

The online version contains supplementary material available at 10.1186/s12877-023-04477-x.

## Background

The continuous progress in medical technology and advancements in living standards have enabled an increasing number of people to live to an advanced age. However, with old age comes a multitude of health issues, and simply living longer does not guarantee a state of good health. According to the World Health Organization (WHO) report, the share of the older population that is above 60 years of age is expected to double between 2015 and 2050, among which nearly 80 percent are from low and middle-income countries [1]. The persistence of communicable diseases, along with an increase in the prevalence of non-communicable diseases, has led to a reduction in the health and well-being of older people. To benefit from the changes in demographics, every nation faces significant challenges in ensuring that its healthcare and social systems are well prepared.

### Health and well-being of the elderly

The absence of sickness is not the only indicator of good health. Health encompasses the aspects of physical, psychological, and social well-being [2]. The researchers given evidence that many older persons categorized as healthy based on the medical tests had vulnerabilities exposed to lifestyle-related or psychological problems, which affected their risks of dying or being handicapped within five years [3]. On the other side, some persons with chronic conditions possessed several qualities that kept them healthy. Overweight older persons with otherwise good physical and mental health, for example, had the lowest chance of dying or becoming handicapped. So, there are different kinds of illnesses faced by older adults. Physical vulnerabilities such as vision problems, loss of hearing, functional disability, chronic obstructive pulmonary diseases, cerebrovascular, cardiovascular diseases, and cancer are common illnesses experienced by old age people all over the world. Apart from these, obesity is another health concern that is affecting an increasing number of seniors around the country. Age-related increases in obesity peak in the 60 s and 70 s increasing the risk of diabetes, arthritis, cardiovascular disease, and several cancer types. According to the Longitudinal Ageing Study of India (LASI), 75% of the elderly suffer from chronic diseases and 40% have a disability due to various factors [4].

The mental health of older is equally significant as their physical health. With the increase in life expectancy, mental illness has significantly influenced the older person’s quality of life. Cognitive deficits, dementia, depression, and anxiety disorders are some of them. It has been pointed out by many researchers that middle- and low-income countries invest a very lesser amount for mental illness and most of it is neglected. At the same time, mental health disease awareness is minimal. Many individuals assume that memory issues and emotions of depression or pessimism are inevitable as they become older, so they delay or avoid getting help. As reported in the World Alzheimer Report 2021, 75% of people with dementia globally are undiagnosed [5]. Many elderly people, particularly those residing in long-term care institutions, are affected by depressive disorders and symptoms. As a result, the demand for mental health treatment in old age is becoming more widespread and urgent.

### Machine learning in geriatric research

Machine Learning (ML) has revolutionized the technology sector with its day-to-day applications. Many developed countries are now adopting this technology to enhance their healthcare service [6]. This raises the question of whether ML will be a powerful tool for enhancing gerontological research. Developing an accurate and quick diagnosis is one of the most difficult aspects of care for geriatric patients. Such individuals bring complicated medical histories and clinical circumstances to healthcare settings, necessitating a focus on how to enhance patient care outcomes for this group. A statistical foundation underpins ML. This should be self-evident, given that ML requires data, which must be characterized using a statistical framework. It allows the user to submit an enormous quantity of data to a computer algorithm, which the computer may then evaluate and make data-driven suggestions and judgments based only on the supplied data [7]. This approach is promising since it readily discovers trends and patterns in a large dataset, is easy to handle multi-dimensional and multi-variety data, has wide applicability in practically every discipline, and is constantly improved.

### Need of the study

Numerous systematic literature reviews have delved into the utilization of machine learning within the realm of geriatric research. Choudhury and colleagues conducted a comprehensive examination of the application of machine learning in geriatric clinical care, and they observed the absence of standardized metrics for evaluating machine learning models, as well as the pressing need for tailored data governance in the healthcare domain [8]. Another literature review explored the integration of machine learning and artificial intelligence in the context of geriatric mental health [9]. This review revealed that dementia stands as the most extensively researched mental health concern within this field, with inconsistent information availability for other mental health issues. Olender et al. undertook a systematic review of studies that used machine learning techniques to predict clinical outcomes in older populations [10], while Leghissa et al. scrutinized research papers focused on the detection, classification, and prediction of frailty in older adults using machine learning methods [11]. The summary of the objective and outcome of these studies have been described in Table 1. In the latter two decades of life, geriatric patients suffer from a variety of ailments, including chronic illnesses, frailty, cognitive decline, and functional dependency. These individuals require high-quality clinical treatment since their issues may lead to hospitalization. As a result, effective solutions for enhancing geriatric clinical care are required. Numerous studies from all over the world have used ML to identify older people at high risk for dementia, predict weakness, risk of falls, pneumonia, delirium, and acute kidney disease, and provide them care. The scope of this study encompasses a comprehensive examination of aging-related concerns, encompassing various facets such as brain age prediction, biological age prediction, chronic diseases, mental health issues, and cognitive disorders. Unlike previous literature, which often concentrated on singular aspects of aging, our research presents a holistic overview, shedding light on the multifaceted aspects of the aging process from creating successful ageing index to using genome data to understand biological ageing, from risk prediction, classification of around all geriatric diseases to multimorbidity, the current study has a broader coverage of literatures in ageing field. The present review mainly focuses on studies that have applied different ML algorithms for accessing the health and well-being and diseases in the elderly population.

**Table 1:** An overview of reviewed studies in the field of geriatrics and machine learning

Study	Objectives	Year	Outcome	Number of studies included in the SLR	Findings
Choudhury et al. [8]	To understand the current use of AI systems, particularly machine learning (ML), in geriatric clinical care for chronic diseases	2020	Individuals over the age of 65 and have one or more chronic illnesses	35 studies	psychological disorder (*n* = 22), eye diseases (*n* = 6), and others (*n* = 7), and the review identified the lack of standardized ML evaluation metrics and the need for data governance specific to health care applications
Olender et al. [10]	To examine the role machine learning in predicting clinical outcomes of older adults	2023	Older adults above age 65 years	37 studies	The meta-analysis indicates that machine learning models display good discriminatory power in predicting mortality
Leghissa et al. [11]	To identify studies based on frailty identification, detection and classification	2023	Frail older adults	41 studies	The data types can be divided into gait data, usually collected with sensors, and medical records, often in the context of aging studies. The most common algorithms are well-known models available from every Machine Learning library
Chowdhury et al. [9]	To identify the current application of machine learning and artificial intelligence in mental health disorders	2021	Studies focusing on electronic health records and administrative health data	21 articles	Electronic health records was the most used data type, and random forest was the most used ML algorithm
Baecker et al. [12]	1. to introduce the reader to the field of brain age prediction and highlight its clinical potential 2. to explain the most common methodological approaches to brain age prediction and discuss five promising clinical applications and possible next steps	2021	brain age prediction	–	Five promising clinical applications of ML- 1. Marker of general brain health, 2. Early detection of brain-based disorders, 3. Prognosis of brain-based disorders, 4. Differential diagnosis of brain-based disorders, 5. Treatment outcome Questions and next steps- 1. Account for inter-scanner heterogeneity, 2. Increase granularity of brain age, 3. Dynamic changes of brain age
Fabris et al. [13]	To review the works that have used supervised ML to study the ageing process	2017	supervised machine learning applied to ageing research	–	The link between specific types of DNA repair and ageing; ageing-related proteins tend to be highly connected and seem to play a central role in molecular pathways; ageing/longevity is linked with autophagy and apoptosis, nutrient receptor genes, and copper and iron ion transport

Note: All the papers have included studies in the review, which have used machine learning methods or artificial intelligence

In the present study, we sought to explore two research questions:

1. What is the current state of research on the application of machine learning in addressing aging-related issues?
2. How has machine learning been applied to study geriatric diseases, the type of population, methods, and datasets used?

So, the objective of the study is to understand the application of ML in solving ageing-related issues by studying the available literature and looking into the more refined measures and methodologies that will show a much better picture of the issue.

## Methods

### Literature search strategy

The systematic review followed the guidelines of the Preferred Reporting Items for Systematic Reviews and Meta-Analysis (PRISMA) [14]. In the current study, older adults are defined as individuals aged 45 and above. The study focuses on the healthy and successful attainment of ageing, with the illness older adults face during this process. PubMed and Scopus databases were used to search original articles, as the authors have access to these databases. The search was conducted focusing on the following three major domains:

- Machine learning
- Geriatric population
- Diseases that occur in old age

For machine learning, we used the keywords Machine Learning, Unsupervised Machine Learning, Supervised Machine Learning. The second set of keywords is Geriatrics, Aged, Older Population for older adults. For the diseases, Diabetes Mellitus, Hypertension, Cancer, Cardiovascular Diseases, Heart Diseases, Lung Diseases, Chronic Obstructive Pulmonary Disease, Alzheimer’s Disease, Parkinson's Disease, Dementia, Mental Health Disorders were used as keywords with OR operator in both PubMed and Scopus for searching papers. Then we combined these three sets of keywords with AND operator to get results for all three domains at once. In the PubMed database, we used the available MeSH (Medical Subject Headings) terms related to our search. Only title, abstract, and keyword sections were selected for the search to get comprehensive and only important literature in the field. The literature search was done on 7th April 2023 and 10th October 2023. The search strategy is clearly explained in the Table 2.

**Table 2:** Search strategy

Domain		**PubMed search query**	**Scopus search query**
#1	Machine Learning	"Machine Learning*"[Mesh] OR "Unsupervised Machine Learning*"[tiab] OR "Supervised Machine Learning*"[tiab]	TITLE-ABS-KEY (( "machine learning" OR "supervised machine learning" OR "unsupervised machine learning")
#2	Geriatric population	((("Geriatrics*"[tiab]) OR "Aged*"[tiab]) OR "Health Services for the Aged*"[tiab]) OR "Frail Elderly*"[tiab]	(elderly* OR "older population" OR geriatric*)
#3	Diseases	(((((((((("Diabetes Mellitus*"[tiab]) OR "Hypertension*"[tiab]) OR "Early Detection of Cancer*"[tiab]) OR "Cardiovascular Diseases*"[tiab]) OR "Heart Disease Risk Factors*"[tiab]) OR "Lung Diseases*"[tiab]) OR "Pulmonary Disease, Chronic Obstructive*"[tiab]) OR "Alzheimer Disease*"[tiab]) OR "Parkinson Disease*"[tiab]) OR "Dementia*"[tiab]) OR "Mental Health*"[tiab]	( "diabetes mellitus" OR hypertension OR cancer OR cardiovascular OR heart OR stroke OR lung OR "pulmonary disease" OR Alzheimer OR "Parkinson's disease" OR "mental health" OR dementia))
Final search query		#1 AND #2 AND #3	#1 AND #2 AND #3

The inclusion criteria of the study were:

- Peer-reviewed papers with available full-text
- Study includes older population and must have focused on any geriatric disease
- Implementation of machine learning algorithm

**Fig. 1:**
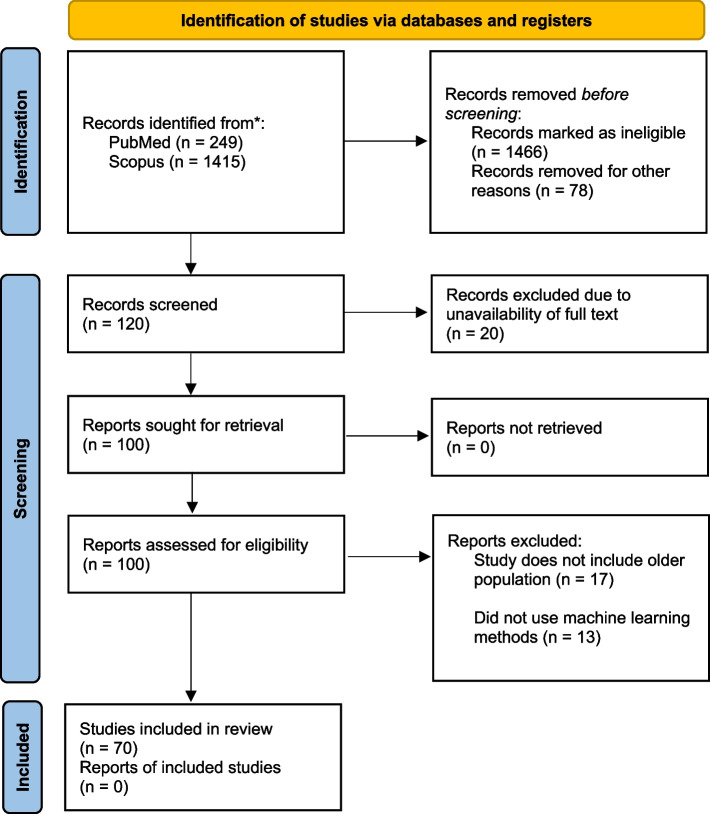
PRISMA Flowchart

Primarily, the research articles were excluded using the filter option of the database, based on the language (only English language articles were selected). We excluded papers focused on only the younger population, or have applied different methodology other than machine learning, and the papers whose full-text version is not available on the web are also excluded from our study. However, we included the studies covering both younger and older populations; and some studies have used deep learning methods, as this method is the advanced application of machine learning methods and is often considered as a subfield of it [15], the studies using only deep learning methods were decided to be included. Both the reviewers (A.D. & P.D.) carefully screened the titles and then abstracts of all the papers and discarded the papers that did not come under the scope of our research and violated our inclusion criteria. The selection of articles was initially done by one reviewer (A.D.) and then checked by another reviewer (P.D.). Figure 1 illustrates the PRISMA flowchart showing the number of articles screened, included, and excluded in each step.

### Data collection process and critical analysis:

Both the reviewers (A.D. & P.D.) studied all the selected articles to collect useful information. The research articles were thoroughly read by one author (A.D.) and the objectives, type of data used and the machine learning methods used were summarised in an Excel sheet, which was further examined by another author (P.D.) to check the correctness.

### Quality assessments of studies

The 70 studies were reviewed for quality by two authors, AD and PD, independently, and arrived at one score with mutual agreement using the Joanna Briggs Institute's (JBI) critical appraisal tool [16]. This tool is commonly employed for evaluating the methodological quality of studies, specifically analytical cross-sectional studies. It comprises eight questions that address issues related to internal validity and the risk of bias, including aspects such as confounding, selection, and the clarity of study sample reporting. A high risk of bias was determined if positive responses were 49% or lower, a moderate risk of bias if the measure fell between 50 and 69%, and a low risk of bias if positive responses exceeded 70%.

## Results

**Fig. 2:**
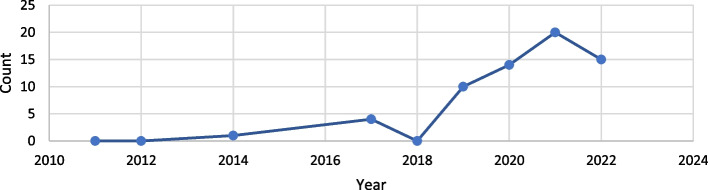
Research papers using ML over the years (from PubMed and Scopus databases)

The use of machine learning in geriatrics and ageing research has been on a significant rise since 2019, which is shown in Fig. 2. In 2021, the number of published papers was the highest compared to any other year.

Initially, we found 249 research articles from the PubMed database and 1415 research articles from the Scopus database, after using the filters according to our inclusion criteria, the number was cut down to 120 peer-reviewed research articles. However, out of these, only 100 papers had free access. After screening the titles of the articles, and screening the abstracts, a total of 70 articles were selected for the critical analysis. The score of the quality assessment of the studies conducted using the Joanna Briggs Institute's (JBI) critical appraisal tool which is available in Table- S2 of the supplementary file. Among the 70 studies, a specific number, only 3 studies showed a moderate risk of bias, while the remaining 67 studies demonstrated a low risk of bias. Importantly, none of the studies included in our review were identified as having a high risk of bias. A summary of the machine learning applications in different geriatric diseases and their objective, datasets, and findings are included in Table 3. Following are the findings from the table, grouped by disease type.

**Table 3:** Summary of studies

SL. No	Author	Year published	Objective	Data set	Outcome	ML methods	Findings	Model performance
1	Kim et al. [15]	2021	1. To compare models employing AI and traditional statistical methods in biological age (BA) prediction using clinical biomarkers 2. To compare the accuracy of BA prediction between various AI models 3. To compare the influence of each clinical biomarker on BA prediction between traditional and AI methods	116,829 subjects aged 20 or older, who received routine health check-ups from 2015 through 2017 community hospitals in Korea	Biological Age	Traditional methods—Linear Regression, 2nd polynomial regression AI, ML methods- XGB regression, RF regression, Support vector regression, Deep neural network	AI models (mainly DNN) produced about 1.6 times stronger linear relationship on average than statistical models, hence outperforming traditional statistical methods in predicting biological age	R- squared value: Linear = 0.84, Polynomial = 0.99, XGBoost = 0.76, Random forest = 0.97, SVR = 0.88, DNN = 0.1
2	Caballero et al. [17]	2017	To create a health score that can be compared across different waves in a longitudinal study, using anchor items and items that vary acorss waves	17,886 subjects from first six ELSA waves	Healthy Ageing/health status	Bayesian multilevel Item Response Theory approach ML methods- Decision Tree, Random Forest	A combination of 2 data anlaytical methdologies was applied to create a measure of health in a cohort, which can be used to understand the ageing process. The metric includes a set of diferent functioning, mobility, sensorineural, cognitive, emotional aspects is feasible and psychometrically sound	NA
3	Qin et al. [18]	2020	To investigate the predictors of the health conditions of older people using machine learning methods	29,477 combined dataset of 2013 & 2015 China Health and Retirement Longitudinal Survey (CHARLS)	Healthy Ageing/health status	Feature selection- Maximal Information Coefficient (MIC), pearson correlation coefficient ML methods- Linear regression, k-nearest neighbors(kNN), XGBoost, Decision Tree, Support Vector Machine, Artificial Neural Network	By extracting non-linear features and establishment of a non-linear model in this experiment, the newly established model is useful to predict the health status of older people. ANN is the best method in terms of acuuracy	Accuracy: Artificial Neural Network = 0.699, Logistic Regression = 0.672, Support Vector Machine = 0.672, XGBoost Classifier = 0.635, Random Forest Classifier = 0.606
4	Engchuan et al. [19]	2019	To evaluate the determinants of health in ageing using machine learning methods and to compare the accuracy with traditional methods	6,209 older adults from 6 waves of ELSA, ALTHOS project	Healthy Ageing/health status	ML models- Random Forest, Deep Learning (ANN), Linear Model	Two models were created 1) A linear model was used to generate the new health metric variable by using sociodemographic variables 2) A health metric prediction model was built by fitting the health metric with the time from previous 4 waves of data. RF was the best performing model	MSE (Mean Square Error): Random Forest = 51.11, Linear Model = 52.07, Deep Learning = 59.08, random prediction = 418.40
5	Wong et al. [20]	2021	To identify and characterise different ageing pathways and associated ageing profiles using multivariate regression trees	25,742 adults from 33 European countries from the fourth European Quality of Life Surveys (EQLS), 2016	Successful Ageing	Unsupervised ML- Multivariate Regression Trees (MRT)	The study identified neighborhood characteristics that contributes to successful ageing and found that healthcare services affordability is a prominently relevant factor	NA
6	Huang et al. [21]	2022	To develop a variety of machine learning models based on psoas muscle tissue at the L3 level of unenhanced abdominal computed tomography to predict osteoporosis	172 adults from hospital (2017 jan to 2021 jan). Collected the CT images and the clinical characteristics data of patients who underwent DXA and abdominal CT examination	Bone Disease	Feature selection- Mann–Whitney U test, LASSO ML models-Gaussian Naïve Bayes (GNB), Random Forest(RF), Logistic Regression(LR), Support Vector Machines(SVM), Gradient Boosting Machine(GBM), XGBoost(XGB)	Gradient Boosting Mahine had the best predictive performance	AUROC (LR = 0.85, XGB = 0.82, GNB = 0.8, GBM = 0.86, RF = 0.87, SVM = 0.81) Sensitivity (LR = 0.73, XGB = 0.7, GNB = 0.73, GBM = 0.7, RF = 0.73, SVM = 0.86), Specificity (LR = 0.86, XGB = 0.75, GNB = 0.86, GBM = 0.92, RF = 0.86, SVM = 0.55), Accuracy (LR = 0.8, XGB = 0.72, GNB = 0.8, GBM = 0.81, RF = 0.8, SVM = 0.71)
7	Birks et al. [22]	2017	To evaluate the risk algorithm derived in Israel on the Clinical Practice Research Datalink (UK)	2550119 patient's primary care electronic health record data	Cancer	Retrospective analysis Model was trained using Israel data and this study tests the model on UK data ML model—Random Forest	The risk score applied to routinely collected primary care data from the UK produced AUC values comparable with those from the Israeli population used to derive it. Age is a crucial factor determining colorectal cancer risk, and the addition of Full Blood Count indices to age and sex improves the identification of patients at risk	NA
8	Sasani et al. [23]	2019	To utilize a machine learning approach to develop an algorithm based on components of the geriatric assessment, other than Timed Up and Go (TUG) test, to accurately predict which patients will have slower TUG times	Electronic medical record—eRFA (1901 patients)	Survival among Cancer Patients	Decision tree classifier	A simple decision tree was able to predict patient gait speed with high accuracy and can be used to screen patients who need further functional assessment or intervention	Accuracy = 78%, Specificity = 90%, Sensitivity = 66% of the prediciton
9	Bosch et al. [24]	2021	1) To predict quality indicators for colorectal cancer surgery 2) To identify previously unrecognized predictors of 30 day mortality, based on a large, nationwide colorectal cancer registry that collected extensive data on comorbidities	62,501 patient's data who underwent resection for primary colorectal cancer registred Dutch ColoRectal Audit	Survival among Cancer Patients	Multivariable Logistic regression, Elasticnet regression, Support vector machine, Random forest, Gradient boosting Risk factors idebtifies by—Logistic regression, Shapley Additive Explanations (SHAP) values	The LR analysis reveals some rare but high-impact comorbidities, such as pulmonary fibrosis, lung surgery or transplant, cardiac valve replacement, and liver failure SHAP analyses revealed that the ASA score and the specific comorbidities of COPD and asthma, hypertension, and myocardial infarction are important variables for predicting postoperative mortality	NA
10	Tseng et al. [25]	2023	1) to validate perviously created biomarkers created for cardiovascular disease (CVD) risk 2) enhance risk assessment in individuals	48,260 subjects with no history of CVD collected clinical data and retinal photographs from UK Biobank	cardiovascular disease	Reti-CVD scores were calculated and stratified into 3 risk groups. Cox proportional hazard models were applied to evaluate the ability of Reti-CVD for predicting CVD	Reti-CVD has the potential to identify individuals with ≥ 10% 10-year CVD risk who are likely to benefit from earlier preventative CVD interventions	NA
11	Huang et al. [26]	2022	To investigate the additive value of four groups of risk factors, based on ease of information availability and regular clinical workflow using ensemble ML	600 Southeast Asian individuals from SingHEART prospective longitudinal cohort study	cardiovascular disease	Ensemble ML for low risk- Naïve Bayes, RF, Support Vector Classifier for high risk- Generalised Linear Regression, Support Vector Regressor, Stochastic Gradient Descent Regressor	The study used novel data sources, i.e., wearable devices data for prediction of CVD risk. Compared the CVD risk score against Framingham Risk Scores and ML algorithm performed better in identifying low risk individuals. Self-reported physical activity, average daily heart rate, awake blood pressure variability and percentage time in diastolic hypertension were important contributors to CVD risk classification	NA
12	Sajid et al. [27]	2021	To develop alternative ML-based risk prediction models (RPMs) that may perform better at predicting CVD status using nonlaboratory features in comparison to conventional RPMs	46 subjects from Punjab Institute of Cardiology, Pakistan through case–control study	cardiovascular disease	ML models- Artificial Neural Networks(ANN), Linear Support Vector Machine(LSVM), Decision Tree (DT)	ML-based RPMs identified substantially different orders of features as compared to baseline RPM. This study concludes that nonlaboratory feature-based RPMs can be a good choice for early risk assessment of CVDs in LMICs. ML-based RPMs can identify better order of features as compared to the conventional approach, which subsequently provided models with improved prognostic capabilities	ANN (AUC = 0.87, accuracy = 81.09, sensitivity = 0.78, specificity = 0.84), Linear SVM (AUC = 0.86, accuracy = 80.86, sensitivity = 0.81, specificity = 0.81), RF (AUC = 0.86, accuracy = 78.30, sensitivity = 0.80, specificity = 0.76)
13	Kobayashi et al. [28]	2022	To identify homogenous echocardiographic phenotypes in community-based cohorts and assess their association with outcomes	827 subjects from STANISLAS to train models 1,394 subjects from Malmö Preventive Project cohort for validation	cardiovascular disease	Cluster analysis-performed K-means clustering based on echocardiograohic data Decision Tree- to find predictive factors	The study identified 3 echocardiographic phenotypes that can be easily identified in clinical practice	NA
14	Barbieri et al. [29]	2022	1) to develop novel deep learning models for predicting the risk of CVD event 2) to compare the performance of the deep learning models and traditional Cox proportional hazards models on the basis of the proportion of explained variance, calibration and discrimination	Study population from New Zealand, 2012	cardiovascular disease	Cox Proportional Hazard Model Deep Learning	The largest hazard ratios estimated by the deep learning models were for tobacco use in women and chronic obstructive pulmonary disease with acute lower respiratory infection in men. Deep learning outperformed Cox proportional hazards models on the basis of proportion of explained variance calibration and discrimination	NA
15	Sanchez-Martinez [30]	2021	To develop a risk prediction model for incident Major Adverse Cardiovascular Events (MACE) from subjects enrolled in a large clinical trial in initially healthy, elderly individuals and to validate the model in a large primary care dataset	ASPirin in Reducing Events in the Elderly (ASPREE) study (18548 participants)	cardiovascular disease	Cox proportional hazard regression models for risk 5 yr risk prediction	A model predicting incident MACE in healthy, elderly individuals includes well-recognised, potentially reversible risk factors and notably, renal function	AUC = 64.16
16	Li et al. [31]	2019	To develop stroke risk classification models based on machine learning algorithms to improve the classification efficiency	National Storke Screening Data, China, 2017	cardiovascular disease	ML models to classify stroke risk levels—Logistic Regression, Naïve Bayes, Bayesian Network Model, DT, Neural Networks, RF < Bagged DT, Voting and Boosting model with DTs	The model developed in this study has capacity to mprove the current screening method in the way that it can avoid the impact of unknown values, and avoid unnecessary rescreening and intervention expenditures	LR (precision = 90.56, recall = 96.35, F1 score = 93.37, AUC = 97.96), NB (precision = 66.96, recall = 94.99, F1 score = 78.55, AUC = 96.64), BN (precision = 67.5 recall = 93.85, F1 score = 78.52, AUC = 96.86), DT (precision = 91.95, recall = 98.12, F1 score = 94.93, AUC = 99.36), NN (precision = 91.82, recall = 98.52, F1 score = 95.05, AUC = 99.23), RF (precision = 96.89, recall = 95.76, F1 score = 96.32, AUC = 99.41), Bagging DT(precision = 92.21, recall = 98.98, F1 score = 95.43, AUC = 99.39), Boosting DT (precision = 94.89, recall = 99.12, F1 score = 96.96, AUC = 99.41)
17	Moradifar et al. [32]	2022	1) to identify socio-economic, life style, and metabolic factors associated with hyperglycemia 2) to compare the ability of 5 commonly used ML algorithms for prediction of hyperglycemia on a population based study	17,705 individuals from Surveillance of Risk Factors of Noncommunicable Disease (STEPs study), Iran 2016	Diabetes Mellitus	ML models-random forest, multivariate logistic regression, gradient boosting, support vector machines, and artificial neural network	Being older age, high BMI status, having hypertension, consuming fish more than twice per week, abdominal obesity were 5 most important risk factors for hyperglycemia. The study shows that survey based screening tools can be used for hyperglycemia prediction, without using blood test	RF (Accuracy = 0.70, specificity = 0.70, sensitivity = 0.68, AUC = 0.70, F1 score = 0.58), XGB (Accuracy = 0.69, specificity = 0.68, sensitivity = 0.72, AUC = 0.70, F1 score = 0.58), SVM (Accuracy = 0.70, specificity = 0.69, sensitivity = 0.70, AUC = 0.70, F1 score = 0.58), LR (Accuracy = 0.70, specificity = 0.70, sensitivity = 0.70, AUC = 0.70, F1 score = 0.58), ANN (Accuracy = 0.69, specificity = 0.69, sensitivity = 0.71, AUC = 0.70, F1 score = 0.58)
18	Chen et al. [33]	2019	To develop a machine learning pipeline to investigate the method’s discriminative value between T2DM patients and normal controls, the T2DM-related network pattern, and association of the pattern with cognitive performance/disease severity	115 subjects from Cross section study &2-year time prospective longitudinal study	Diabetes Mellitus	Ml methods-Principal Component Analysis, feature selection, logistic regression classifier	The machine learning based method is superior to the widely-used univariate group comparison method. The individual perfusion diabetes pattern score is a highly promising perfusion imaging biomarker for tracing the disease progression of individual T2DM patients	NA
19	Mansoori et al. [34]	2023	To assess the potential association between T2DM and routinely measured hematological parameters	9000 adults from Mashhad stroke and heart atherosclerotic disorder (MASHAD) cohort study	Diabetes Mellitus	ML models-logistic regression, decision tree, Bootstrap Forest (BF)	BF model performed the best. The most effective factors in the BF model were age and WBC (white blood cell). The BF model represented a better performance to predict T2DM	NA
20	Lai et al. [35]	2019	1) to build an effective predictive model using ML to identify the population at risk of diabetes mellitus based on lab information and demographic data of patients 2) to compare the predictability of ML models	13,309 Canadian patients from Canadian Primary Care Sentinel Surveillance Network (CPCSSN)	Diabetes Mellitus	ML models -logistic regression, Gradient Boosting Machine (GBM), decision tree, random forest	The GBM and Logistic Regression models perform better than the Random Forest and Decision Tree models. Fasting blood glucose, body mass index, highdensity lipoprotein, and triglycerides were the most important predictors in these models	AROC (GBM = 84.7%, Logistic Regression = 84.0%, Random Forest = 83.4%, RPART = 78.2%)
21	Li et al. [36]	2021	To use ML to select sleep and pulmonary measures associated with hypertension development	Prospective cohort study 860 individuals from Sleep Heart Health Study (SHHS), 261 developed hypertension after 5 years	Hypertension	Penalized Regression	A unique combination of sleep and pulmonary function measures (using ML) better predicts hypertension compared to the apnoea-hypopnea index	NA
22	Zhong et al. [37]	2023	To develop a superior ML model based on easily collected variables to predict the risk of early cognitive impairement in hypertension individuals	Multicenter observational study including 733 hospitalized hypertensive patients	Hypertension	Feature Selection -LASSO regression ML classifiers—Logistic Regression, XGBoost, Gausian Naïve Bayes	Hip circumference, age, education levels, and physical activity were considered significant predictors of early cognitive impairment in hypertension XGBoost performed best	LR (AUC = 0.83, accuracy = 0.74, sensitivity = 0.78, specificity = 0.73, F1 score = 0.50), XGB (AUC = 0.88, accuracy = 0.81, sensitivity = 0.84, specificity = 0.80, F1 score = 0.59), GNB (AUC = 0.74, accuracy = 0.75, sensitivity = 0.74, specificity = 0.74, F1 score = 0.50)
23	Sun et al. [38]	2022	To investigate the association between waist circumference and the development of hypertension	5,330 individuals from CHARLS	Hypertension	Adjusted Cox Regression Model and visualized by restricted cubic splines, sensitivity analysis of cox regression in different subgroups ML models—Random Forest, XGBoost	The random forest method and the Extreme Gradient Boosting method revealed waist circumference as an important feature to predict the development of hypertension. The sensitivity analysis indicated a consistent trend between waist circumference and new‐onset hypertension in all BMI categories	NA
24	Alkaabi et al. [39]	2020	To construct and compare predictive models to identify individuals at high risk of developing hypertension without the need of invasive clinical procedures	987 individuals from Qatar Biobank study	Hypertension	ML models-Decision Tree, Random Forest, Logistic Regression	In terms of AUC, compared to logistic regression, random forest and decision tree had a significantly lower discrimination ability. Age, gender, education level, employment,tobacco use, physical activity, adequate consumption offruits and vegetables, abdominal obesity, history ofdiabetes, history ofhigh cholesterol, and mother’s history high blood pressure were important predictors ofhypertension	LR (accuracy = 81.1%, PPV = 80.1%, sensitivity = 81.1%, F measure = 80.3%), DT (accuracy = 82.1%, PPV = 81.2%, sensitivity = 82.1%, F measure = 81.4%), RF (accuracy = 82.1%, PPV = 81.4%, sensitivity = 82.1%, F measure = 81.6%)
25	Alghafees et al. [40]	2022	To use machine learning to predict the stone free stauts after percutaneous nephrolithotomy (PCNL) among patients	137 patients who ahave undergone PCNL at a hospital of Soudi Arabia. A 12 month followup study was done between 2020–2022	Kidney Disease	Supervised ML-Logistic regression, Random forest, XGBoost regressor	A inverse relation was found between Stone Free Status and Chronic Kidney disease and Hypertension. Random forest model showed the highest efficacy in predictingstone free status	Accuracy: LR = 71.4%, XG Boost = 74.5%, RF = 75%
26	Jadlowiec et al. [41]	2022	To better understand differeing delayed graft function (DGF) outcomes, clustering approach was used to categorize clinical phenotypes of Kidney Transplant recepients with DGF and their paired donors	17,073 patients who received kidney transplant in the USA (2015–19) were identified using Organ Procurement and Transplantation/ United network for Organ Sharing database	Kidney Disease	Estimation of cumulative risks of death censored graft failure and death after kidney transplant—Kaplan–Meier analysis Comparision of risk among assigned clusters—Cox proportional hazaed analysis Clustering (ML)—Unsupervised consensus clustering approach	By applying a ML clustring approach, the current study has allowed for an unbiased assessment of kidney transplant outcomes for those with DGF 4 clusters were characterized: cluster 1- younger, low BMI, non-diabetic, kidney re-transplant recipients who had a high PRA (panel reactive antibody). cluster2—oldest of the four clusters, had a higher BMI, were likely to have lower functional status, and be diabetic with 3 + years of dialysis vintage. They were also the most likely to receive ECD (extended croterion donor) high KDPI (kidney donor profile index) kidneys. cluster 3—young and non-diabetic. They were more likely to be black, have hypertension and receive higher HLA mismatched, lower KDPI kidneys. cluster 4—middle-aged, firsttime KT recipients with either diabetes or hypertension, lower functional status, dialysis duration ≥ 3 years, and a low PRA	NA
27	Sabanayagam et al. [42]	2020	To detect chronic kidney disease from retinal images using deep learning algorithm (DLA)	3 population based, multiethnic, cross-sectional studies from Singapore and China. For deleopment and validation of DLA—Singapore epidemiology of eye diseases study(SEED) For ecternal testing—Singapore prospective study program (SP2), Beijing eye study (BES)	Kidney Disease	3 models were trained- 1) image DLA 2) risk factors (RF) including age, sex, ethnicity, diabetes and hypertension 3) hybrid DLA combining image and RF	The image-only DLA and clinical RF models both achieved high AUCs in SEED internal validation. The performance of image DLA in subgroups of patients with diabetes and hypertension was similar to that in the whole group. Thus, for chronic kidney disease detection, a retinal image-only DLA is similar to information from a classic RF model, and supports the potential of using retinal photography to detect chronic kidney disease in specific settings	AUC, sensitivity, specificity, PPV, NPV were reported for image only, RF, only and hybrid differently for the datasets
28	Cho et al. [43]	2021	To develop a model for predicting suicide among elderly popoulation by using ML	48,047 South Korean elderly data from National Health Insurance Sharing Service (NHISS)	Mental Health	ML model—Random Forest	The study developed a model for predicting suicide that occurs infrequently using ML. The suicide group had a more prominent history of depression, with the use of medicaments significantly higher. Body mass index, waist circumference, total cholesterol, and low-density lipoprotein level were lower in the suicide group	Random Forest (AUC = 0.818, accuracy = 0.832, sensitivity = 0.600, specificity = 0.833, NPV = 0.999, PPV = 0.007)
29	Kasthurirathne et al. [44]	2019	To build decision models caapble of predicting the need of advanced care for depression across patients	84,317 individuals from Primary Care Visit at Eskenazi Health, Indiana	Mental Health	ML model—Random forest decision models	This study demonstrates the ability to automate screening for patients in need of advanced care for depression across (1) an overall patient population or (2) various high-risk patient groups using structured datasets covering acute and chronic conditions, patient demographics, behaviors, and past visit history	AUC, optimal sensitivity, optimal specificity was reported for different patient groups
30	Zhang et al. [45]	2022	To explore the spatial patterns of urban streetscape features and their associations with residents’ mental health by age and sex in Zhanjiang, China	Study area where images are captured-Zhanjiang City, China Mental health data—813 patients suffering psychiatric disorders from hospitalization data of Guangdong Medial University	Mental Health	Image capturing- Baidu Street View physical features- Green View Index (GVI) Spatial distributions- Global Moran's I and hotspot analysis Deep learning methods—Fully Convolutional Network for semantic image segmentation	The Results of Pearson’s correlation analysis show that residents’ mental health does not correlate with GVI, but it has a significant positive correlation with the street enclosure, especially for men aged 31 to 70 and women over 70-year-old	NA
31	Opoku et al. [46]	2021	1) to investiagate the feasibility of predicting depression with human behaviours quantified from smart phone datasets 2) to identify behaviours that can influence depression	Data of 629 participants collected in a longitudinal observational study with the Carat app in 6 months interval Smart phone datsets and self-reported 8-item Patient Health Questionnaire depression assessments	Mental Health	the relationship between the behavioral features and depression—correlation and bivariate linear mixed models (LMMs) ML models- RF, SVM with radial basis function kernel, XGBoost, KNN, Logistic Regression	Our findings demonstrate that behavioral markers indicative of depression can be unobtrusively identified from smartphone sensors’ data. Screen and internet connectivity features were the most influential in predicting depression	RF (accuracy = 97.97, precision = 92.50, recall = 94.38, F1 = 93.41, AUC = 98.83, cohen kappa = 92.21), XGB (accuracy = 98.14, precision = 92.51, recall = 95.56, F1 = 94.0, AUC = 99.06, cohen kappa = 92.90), SVM (accuracy = 85.68, precision = 51.98, recall = 80.67, F1 = 63.20, AUC = 89.47, cohen kappa = 54.83), LR (accuracy = 59.27, precision = 20.29, recall = 57.25, F1 = 29.95, AUC = 62.43, cohen kappa = 9.66), KNN (accuracy = 96.44, precision = 85.55, recall = 92.19, F1 = 88.73, AUC = 94.69, cohen kappa = 86.61)
32	Ewbank et al. [47]	2020	1) to generate a quantifiable measure of psychotherapy treatment 2) to determine the association between the quantity of each aspect of therapy delivered and clinical outcomes	17,572 patients receiving cognitive behavioural therapy (CBT) collected through internet	Mental Health	Multivariable Logistic Regression, for classification—Deep Learning	The finding supports the principle that CBT change methods help produce improvements in patients presenting symptomps. Deep leaning allows us to extract this knowledge to provide valuable insights into therapy that were previously unavailable to an individual therapist	NA
33	Guntuku et al. [48]	2019	1) to characterise the lives of people who mention the wordd 'lonely' and 'alone' in their Twitter timeline 2) to correlate their posts with predictors of mental health	6202 Twitter users who used 'lonely' and 'alone' in their posts in the timeline 2012 to 2016 Control group matched by age, gender and period of acitivity	Mental Health	Language features extracted by- Open vocabulary, Dictionary based, Mental well-being attributes, Use of drug words, Temporal patterns For predicitng the likelihood of posting (ML model)—Random Forest	The cases of loneliness attributed to difficult interpersonal relationships, psychosomatic symptopms, substance use, wanting change, unhealthy eating and having troubles with sleep. These posts were also aqssociated with linguistic markers of anger, depression and anxiety The Random Forest model predicted expression of lonliness with around 86% accuracy score	AUC, F1 score, accuracy, precision, recall values are given for different features
34	Helbich et al. [49]	2019	1) to compare streetscape metrics derived from street view images with statellite-derived ones for the assessment of green and blue space 2) to examine associations between exposure to green and blue spaces as well as geriatric depression	Questionnaire based cross-sectional study of 1190 individuals Image data- streetview images and satellite images	Mental Health	Normalized Difference Vegetation Index (NDVI), and Normalized Difference Water Index (NDWI) were analyzed using a fully convolutional Neural Network Depressive symptoms were assessed through the shortened Geriatric Depression Scale (GDS-15) and analyzed by Multilevel Regressions	Metrics of green and blue space derived from street view images were not correlated with satellite-based ones Multilevel regressions showed that both street view green and blue spaces were inversely associated with GDS15 scores No significant associations were found with NDVI, NDWI, and GlobeLand30 green and blue space	NA
35	Kim et al. [50]	2021	1) To classify and predict associations between nutritional intake and risk of overweight/ obesity, dyslipidemia, hyeprtension and type 2 diabetes mellitus (T2DM) 2) To develop a deep neural network (DNN) model and compare it with the machine learning models	4th to 7th Korea National Health and Nutrition Examination Survey (KNHANES) samples: dylipidemia = 10,731 hypertension = 10,991 T2DM = 3,889 overweight/obesity = 10,980	Multimorbidity	DNN (binary cross entropy loss funtion for binary classification) Stuctural Equation Modeling performed to simultaneously estimate multivariate causal association between nutritional intake and the specified diseases. ML models for comparision—logistic regression, decision tree	DNN has better prediciton accuarcy than 2 conventional machine learning models. Energy intake was the most influential factor in risk of dyslipidemia, hypertension and overweight/obesity. (here, Nutritional intake includes food intake, energy intake, protein intake, fat intake, carbohydrate intake, sodium intake and potassium intake)	Accuracy according to diseases: Dyslipidemia (DNN = 0.58654, LR = 0.58448, DT = 0.52148), Hypertension (DNN = 0.79958, LR = 0.79929, DT = 0.66773), T2DM (DNN = 0.80896, LR = 0.80818, DT = 0.71587), Overweight/obesity (DNN = 0.62496, LR = 0.62486, DT = 0.54026)
36	Sone et al. [51]	2022	To investigate the relationships between brain aging and relevant mental factors as well as lifestyle-related metabolic diseases in a cognitively unimpaired population of older participants	community-based cohort study (773 participants)	Multimorbidity	Multiple regression analysis	The analysis identified life satisfaction, diabetes, and use of alcohol as significantly independent predictors for brain age in a community-based elderly cohort. Resilience may also be important. It is possible that people could keep their brains younger by improving their subjective life satisfaction, avoiding alcohol use disorder, and preventing the development of diabetes	NA
37	Byeon et al. [52]	2021	To examine Alzheimer's patients living in South Korea to understand the predictors of anxiety using boosting algorithms and data-level approach and compare the performance	Rehabilitation hospitals for early dementia screening (253 individuals)	Multimorbidity	Boosting algorithms (AdaBoost and XGBoost) Data-level approach (raw data, undersampling, oversampling, and SMOTE)	Using a SMOTE-XGBoost model may provide higher accuracy than using a SMOTE-Adaboost model for developing a prediction model using outcome variable imbalanced data such as disease data in the future	XGBoost based on SMOTE (accuracy = 0.84, sensitivity = 0.85, and specificity = 0.81)
38	Mahajan et al. [53]	2021	To develop a physiologically diverse and generalizable set of multimorbidity risk score	9,92,868 patient's records from a 2 year followup electronic health record	Multimorbidity	Built 6 ML models for scoring risk of heart, lung, neuro, kidney, digestive functions and a combined score for all ML models- Logistic regression, gradient boosted tree classifier	The total health score (THS) created in this study, outperformed other scores created by using conventional methods (Charlson comorbidity index and Elixhauser comorbidity index). The performance of the newly created score was most accurate for middle aged, lower-income subgroups	Total health score (AUROC = 0.823, sensitivity = 0.721, specificity = 0.777) Also, AUROC < sensitivity, specificity scores are given for all the diseases
39	Spooner et al. [54]	2020	To develop ML models that predict survival to dementia using baseline data from different studies	Longitudinal ageing studies- Sydney Memory and Ageing Study (MAS), Alzheimer’s Disease Neuroimaging Initiative (ADNI)	Neurodegenrative Disease	8 feature selectio n methods: Filter methods- univariate cox score, RF variable importance, RF minimal depth, RF variable hunting, RF maximally selected rank statistics,mRMR Wrapper methods- sequential forward selection, sequential forward floating selection. ML models- LASSO, Ridge, ElsticNet regression, CoxBoost, GLMBoost, XGBoost, random survival forest, maximally selected rank statistics from random surival forest. evaluation metric- c index	Ridge regression outperformed LASSO in all personalised Cox regression methods Among the boosted models, CoxBoost was the best performer. The maximally selected rank statistics RF outperformed the other random forest models. In case of feature selection, RF min depth filter produced most accurate models	NA
40	Tan et al. [55]	2023	To develop a reliable ML model using socio-demographics, vascular risk factors, and structural neuroimaging markers for early diagnosis of cognitive impairement in multi-ethnic Asian population	911 participants from Epidemiology of Dementia in Singapore study	Neurodegenrative Disease	ML models- logistic regression, support vector machine, gradient boosting machine voting ensemble- SHAP	According to the voting ensemble, the important predictors of cognitive impairement are age, ethnicity, education attainment, and structural neuroimaging	LR (accuracy = 0.71, F1 = 0.78, AUC = 0.69, FPR = 0.38, sensitivity = 0.75, specificity = 0.62, PPV = 0.81, NPV = 0.54), SVM (accuracy = 0.74, F1 = 0.81, AUC = 0.71, FPR = 0.40, sensitivity = 0.81, specificity = 0.60, PPV = 0.81, NPV = 0.59), GBM (accuracy = 0.73, F1 = 0.79, AUC = 0.73, FPR = 0.29, sensitivity = 0.74, specificity = 0.71, PPV = 0.85, NPV = 0.56), Ensemble (accuracy = 0.83, F1 = 0.87, AUC = 0.80, FPR = 0.26, sensitivity = 0.86, specificity = 0.74, PPV = 0.88, NPV = 0.72)
41	Hu et al. [56]	2021	To build a prediction model based on ML for cognitive impairement among Chinese community dwelling elderly people with normal cognition	6718 individuals of age > 60, with MMSE score > = 18, not having any severe disease from the Chinese Longitudinal Health Longevity Survey (CLHLS)	Neurodegenrative Disease	To access 3-year risk of developing cognitive impairement, Ml models used- Random forest, XGBoost, Naïve Bayes, Logistic regression A nomogram was established to vividly present the prediction model	Features selected to develop model- age, instrumental activities of daily living, marital status, and baseline cognitive function Older people with nomogram score less than 170 are considered to have a low 3-year risk, and more than 173 are considered at higher risk	AUC, optimal cut off, sensitivity, specificity, accuracy, specificity/sensitivity values were reported for logisitc regression, random forest, naïve bayes, XG Boost both for validation dataset and test dataset
42	Fukunishi et al. [57]	2020	To predict the risk of Alzheimer-type dementia for persons aged over 78 without receiving long-term care services using regularly collected claim data	48,123 persons from claim data of health insurance and long-term care insurance in Japan	Neurodegenrative Disease	ML models- Sparse logistic regression models with L0, L1 regularization	SLR-L0 is more effective than SLR-L1 in dealing with a large number of features and useful for practical use. It can be extended to prediction of various diseases	SLR-L0 (Accuracy = 0.639, Precision = 0.105, Recall (Sensitivity) = 0.617, Specificity = 0.641, False positive rate = 0.359, False negative rate = 0.383, F-measure = 0.180, AUC = 0.663, Average precision = 0.124), SLR-L1 (Accuracy = 0.623, Precision = 0.103, Recall (Sensitivity) = 0.633, Specificity = 0.622, False positive rate = 0.378, False negative rate = 0.367, F-measure = 0.177, AUC = 0.660, Average precision = 0.122)
43	Shi et al. [58]	2021	To analyze the relationship between ageing, cellular homeostasis and Neurodegenrative diseases, as well as the relative mechanism involced	DNA methylation profiles obtained from Gene Expression Omnibus (GEO) database	Neurodegenrative Disease	Feature selection- ReliefF Supervised ML	The extracellular fluid, cellular metabolisms, and inflammatory response were identified as the common driving factors of cellular homeostasis imbalances during the accelerated aging process	NA
44	Lian et al. [59]	2020	To identify and classify Alzheimer's disease using a novel Weakly supervised learning based deep learning (WSL- based DL) framework	2 brain MRI datasets 2D MRIand #D MRI data) one from Kaggle	Neurodegenrative Disease	WSL-based deep learning (DL) framework (ADGNET)	The ADGNET has higer F-score and sensitivity value, outperforming two state of art models (ResNext WSL and SimCLR)	Kappa score, sensitivity, specificity, precision, accuracy, F1 score values are reported for all the models and datasets
45	Szlejf et al. [60]	2023	To develop and test ML models to predict cognitive impairement using variables obtainable in primary care settings,	8,291 participants from a cross-sectional study ELSA-Brasil	Neurodegenrative Disease	ML models- Logistic regression, neural networks, gradient boosted trees	XGBoost presented the highest discrimination in predicting cognitive impairement than the other models. Seventy-six percent of the individuals with cognitive impairment were included among the highest ranked individuals by this algorithm	XGBoost 0.873, ROC-AUC = 0.316, sensitivity = 0.969, specificity = 0.298, PPV = 0.972, NPV = 0.307 76.53% LightGBM 0.860 (0.821–0.898) 0.398 0.967 0.331 0.975 0.361 72.44% Logistic Regression 0.805 (0.762–0.847) 0.235 0.964 0.209 0.969 0.221 61.22% ANN 0.801 (0.755–0.845) 0.204 0.967 0.200 0.967 0.202 66.32% Catboost 0.805 (0.762–0.847) 0.102 0.989 0.270 0.964 0.148 61.22%
46	Benhamou et al. [61]	2021	Hypothesis 1- Frontotemporal dementia syndromes would be associated with more severe impairments of musical deviant detection and autonomic reactivity than would Alzheimer’s disease. Hypothesis 2- Sensitivity to information-theoretic parameters of melodies (deviant surprise, melody entropy) would be relatively more severely reduced in bvFTD and svPPA than in other participant groups Hypothesis 3- Cognitive coding of musical surprise in the patient cohort would have separable neuroanatomical correlates within the hierarchical distributed brain networks previously implicated in processing different kinds of musical information	case- 62 patients with frontotemporal dementia, typical amnestic Alzehimer's disease control- 33 healthy persons	Neurodegenrative Disease	Regression model for that took elementary perceptual, executive and musical competence into account, assessed accuracy detecting melodic deviants And pupillary responses and related these to deviant surprise value and carrier melody predictability, calculated using an unsupervised ML model of music	Major dementias have distinct profiles of sensory ‘surprise’ processing, as instantiated in music Music may be a useful and informative paradigm for probing the predictive decoding of complex sensory environments in neurodegenerative proteinopathies, with implications for understanding and measuring the core pathophysiology of these diseases	XG Boost (AUC-ROC = 0.87, sensitivity = 0.31, specificity = 0.96, PPV = 0.29, NPV = 0.97, F1 score = 0.30), LightGBM (AUC-ROC = 0.86, sensitivity = 0.39, specificity = 0.960.96, PPV = 0.33, NPV = 0.97, F1 score = 0.36), LR (AUC-ROC = 0.80, sensitivity = 0.23, specificity = 0.96, PPV = 0.20, NPV = 0.96, F1 score = 0.22), ANN (AUC-ROC = 0.80, sensitivity = 0.20, specificity = 0.96, PPV = 0.20, NPV = 0.96, F1 score = .20), Catboost (AUC-ROC = 0.80, sensitivity = 0.10, specificity = 0.98, PPV = 0.27, NPV = 0.96, F1 score = 0.14)
47	Ithapu et al. [62]	2014	To detect and quantify White Matter Hyperintensities (WMH) observed in T2 FLAR images of subjects with risk of neurological disorders, especially Alzheimer's disease	T1 and T2-MRI scans of 251 individuals from Wisconsin Alzheimer's Disease Research Center	Neurodegenrative Disease	ML models- Random forests, Support Vector machines	Random Forest based regression works the best with significant improvement over the current state-of-the-art unsupervised model	SVM (F = 0.54, precision = 0.56), RF (F = 0.67, precision = 0.79)
48	Gharbi-Meliani et al. [63]	2023	1) to built a clustering analysis for identifying transition to high likelihood dementia in population ageing surveys 2) to demostrate that the suggested model can identify probable dementia in surveys where dementia is wither poorly or non-diagnosed, and that the method is also efficient to study the risk factors	For model building- wave 1 & 2 of Survey of Health, Ageing, and Retirement in Europe (SHARE) validation set- English Longitudinal Study of Ageing (ELSA) waves 1–9 Harmonised datasets from the Gateway to Global Aging	Neurodegenrative Disease	The discrimination power of the proposed clustering algorithm was evaluated by counting on its identification of "likely dementia" status compared with the self-reported dementia status Unsupervised ML—Multiple Factor Analysis (MFA) followed by Hierarchical Clustering on Principal Components (HCPC)	“Likely Dementia” status was more prevalent in older people, displayed a 2:1 female/male ratio and was associated with nine factors that increased risk of transition to dementia: low education, hearing loss, hypertension, drinking, smoking, depression, social isolation, physical inactivity, diabetes, and obesity. Results were replicated in ELSA cohort with good accuracy	NA
49	Ford et al. [64]	2019	To detect existing or developing dementia on patients which is currently undetected as having the condition by the general practice	case–control design 93,120 patients from electronic patient records from Clinical Practice Research Datalink (CPRD)	Neurodegenrative Disease	ML classifiers to discriminate between cases and controls—logistic regression with lasso, naïve bayes, support vector machines, random forest, neural network	The top features retained in the logistic regression model were disorientation and wandering, behaviour change, schizophrenia, self-neglect, and difficulty managing. Naïve Bayes model performed least well Logistic regression and random forest algorithms may nevertheless offer an advantage over support vector machines and neural networks as they produce easy to interpret	Logistic Regression with Lasso (AU-ROC = 0.736, specificity = 0.752, sensitivity = 0.602, PPV = 0.156), Naïve Bayes (AU-ROC = 0.682, specificity = 0.906, sensitivity = 0.241, PPV = 0.164), SVM (AU-ROC = 0.737, specificity = 0.691, sensitivity = 0.674, PPV = 0.142), RF (AU-ROC = 0.734, specificity = 0.653, sensitivity = 0.700, PPV = 0.134), NN (AU-ROC = 0.737, specificity = 0.781, sensitivity = 0.619, PPV = 0.178)
50	Casanova et al. [65]	2020	To evaluate modifiable and genetic risk factors for Alzheimer's disease to predict cognitive decline	7,142 paricipants with DNA and > 2 cognitive evaluations from HRS (Health and Retirement Study)	Neurodegenrative Disease	To determine the form and number of classes- Latent class trajectory analysis ML model- Random forest classifier	Top ranked predictors were education, age, gender, stroke, NSES, and diabetes, APOE ε4 carrier status, and BMI RF classification techniques suggested that nongenetic factors contribute more to cognitive decline than genetic factors. Education was the most relevant predictor for discrimination	NA
51	Aguayo et al. [66]	2023	To compare the performance of different types of deep neural networks (DNNs) with regularized Cox proportional hazard models to predict neurodegenrative diseases in older population	5433 participants having no neurodegenerative condition from wave 2 & wave 8 for followup from ELSA study	Neurodegenrative Disease	Outcome- new events of Parkinson's, Alzheimer's or Dementia. Models- DNNs- Feedforward, TabTransformer, Densenet Cox models—CoxEn, CoxSf	TabTransformer is promising for prediction of neurodegenrative diseases with heterogeneous tabular datasets with numerous features. Moreover, it can handle censored data. However, Cox models perform well and are easier to interpret than DNNs. Therefore, they are still a good choice for neurodegenrative diseases	NA
52	Oscar et al. [67]	2017	To develop and demonstrate a supervised method for coding the content of sample of tweets on several dimensions relevant to Alzheimer's disease stigma on social media platform (twitter)	31,150 tweets related to Alzheimer's disease (AD) collected through Twitter's search API 9 AD related keywords were searched	Neurodegenrative Disease	Tweets were coded into 6 dimensions- informative, joke, metaphorical, organization, personal experience, ridicule. Classifier- N-grams Content analysis- Linguistic Inquiry and Word Count (LIWC)	The study identified that 21.13% of the AD-related tweets used AD-related keywords to perpetuate public stigma, which could impact stereotypes and negative expectations for individuals with the disease and increase “excess disability”	NA
53	König et al. [68]	2021	To investigate the association between automatically extracted speech features and neuropsychiatric symptomps (NPS) in patients with mild NPS	141 patients with NPS. From hospital records	Neurodegenrative Disease	A clinical score NPI (neuropsychiatric inventory) was used for the assessment. ML models- Support vector regression, Lasso linear regression	Machine learning regressors are able to extract information from speech features and perform above baseline in predicting anxiety, apathy, and depression scores. Different NPS seem to be characterized by distinct speech features, which are easily extractable automatically from short vocal tasks	NA
54	Prange and Sonntag [69]	2022	To use digital pen features, such as geometrical, spacial, temporal and pressure characteristics to model user's cognitive performance (binary classification)	40 subjects from a geriatric daycare clinic	Neurodegenrative Disease	Traditional approach—content analysis of drawn features Current approach- digital cognitive assessment ML models- SVM, LR, nearest neighbors, naïve bayes, DT, RF, AdaBoost, Gradient boosted trees, deep learning	ML techniques our feature set outperforms all previous approaches on the cognitive tests considered, i.e., the Clock Drawing Test, the Rey-Osterrieth Complex Figure Test, and the Trail Making Test in a binary classification task	Accuracy, F1 score, Log loss, Precision, Recall, AUC was calculated for feature subsets
55	Younan et al. [70]	2020	1) to examine whether PM2.5 (particulate matter) affects the episodic memory decline, 2) to explore the potential mediating role of increased neuroanatomic risk of Alzheimer’s disease associated with exposure	531 older females from Women's Health Initiative Study of Cognitive Ageing & the Women's Health Initiative Memory Study of Magnetic Resonance Imaging (1999–2010)	Neurodegenrative Disease	Subjects were assigned Alzheimer's disease pattern similarity scores through brain MRI. Method applied- Structural Equation Modeling (SEM)	The continuum of PM2.5 neurotoxicity that contributes to early decline of immediate free recall/new learning at the preclinical stage, which is mediated by progressive atrophy of grey matter indicative of increased Alzheimer’s disease risk, independent of cerebrovascular damage	NA
56	Aschwanden et al. [71]	2020	To estimate the relative importance of selected predictors in forecasting cognitive impairement and dementia in a large scale population representative sample	9,979 older adults from HRS	Neurodegenrative Disease	Combined methodology Estimatinf relative importance- RF and survival analysis estimate effect size for imp vars- Cox proportional hazard model	African Americans and individuals who scored high on emotional distress were at relatively highest risk for developing cognitive impairment and dementia. Lower education, Hispanic ethnicity, worse subjective health, increasing BMI were comparatively strong predictors for cognitive impairment	NA
57	Noh et al. [72]	2021	To use machine learning (ML) to identify important features of gait and physical fitness to predict a decline in global cognitive function in older adults	306 older adults from a survey conducted in Busan, South Korea	Neurodegenrative Disease	Feature ranking- simple linear regression, XGBoost ML models- SVM, DT, RF, Neural Network, LASSO, ElasticNet, SCAD, MCP	Five optimal features were selected using elastic net on the LP data for men, and twenty optimal features were selected using support vector machine on the XI data for women. Thus, the important features for predicting a potential decline in global cognitive function in older adults were successfully identified	NA
58	Jia et al. [73]	2020	To identify variables associated with subsequent incident dementia using ML	1,439 individuals from Monongahela-Youghiogheny Healthy Aging Team (MYHAT) cohort study (2006–08)	Neurodegenrative Disease	ML method- Markov modeling with Hybrid density-and partition-based (HyDaP) clustering	The probability of incident dementia was associated with worse self-rated health, more prescription drugs, subjective memory complaints, heart disease, cardiac arrhythmia, thyroid disease, arthritis, reported hypertension, higher systolic and diastolic blood pressure, and hearing impairment, depressive symptoms, not currently smoking, and lacking confidantes	NA
59	Garcia et al. [74]	2019	To investigate whether early behavioural signs of AD may be detected through dialogue interaction	Middle aged participants from PREVENT Dementia Study, 2015	Neurodegenrative Disease	Proposed methods-Speech processing ML methods- linear generative classifiers, state-of-art deep architectures	The study introduced a novel approach to monitoring early signs of dementia through the analysis of spoken dialogue. Also, focused more on narrative speech (monologue), both from transcribed recordings and from signal processing of voice samples	NA
60	Liu et al. [75]	2023	1) to explore the predictive value of machine learning in cognitive impairement, 2) to identify important factors for cognitive impairement	2,326 older adults from baseline, year2, year4 followups from CHARLS (2011–2015) data	Neurodegenrative Disease	ML models for predicting cognitive impairement- Random Forest, Logistic Regression	Random forest models showed high accuracy for all outcomes at Year 2, Year 4, and cross-sectional Year 4. BMI, Blood pressure, cholesterol, functioning functional limitations, age, and depression were identified as important predictors of cognitive impairment	Precision, recall, F score, accuracy of different models of RF anf LR are reported for cognitive impairement prediciton for different time period
61	Elgammal et al. [76]	2022	To propose a novel computational method to automatically classify various stages of Alzheimer’s Disease based on the utilization of multifractal geometry analysis	Kaggle (560 MRI) Alzheimer's Disease Neuroimaging Initiative (ADNI) database (750 MRI images)	Neurodegenrative Disease	Multifractal analysis K-nearest neighbour algorithm, XG Boost, Random Forest	The proposed technique has achieved 99.4% accuracy and 100% sensitivity and the comparative results show that the proposed classification technique outperforms is recent techniques	XG Boost (accuracy = 73.2%, F1 score = 21.4%, ROC-AUC = 53.0%), RF (accuracy = 82.7%, F1 score = 83.30%, ROC-AUC = 82.70%)
62	Ghazal et al. [30]	2021	To classify multiple Alzheimer's disease stages using a novel methodology i.e. transfer learning	Kaggle repository (6393 image samples)	Neurodegenrative Disease	Transfer learning (Alexnet, a deep learning based network)	The proposed algorithm used the pertained AlexNet for the problem, retrained the CNN, and validated on validation dataset which gave an accuracy of 91.7% for multi-class problems on 40 epochs and the proposed system model does not require any hand-crafted features and it is fast or can easily handle small image datasets	The proposed model has accuracy of 73.75%
63	Sountharrajan et al. [77]	2022	To classify the patient records with dementia and non-dementia using different machine learning techniques from MRI brain images	Open Access Series of Imaging Studies (OASIS-2) dataset (150 patients)	Neurodegenrative Disease	logistic regression, Decision Tree (DT) classification, Random Forest (RF) classification, Support Vector Machine (SVM) classification and AdaBoost Classification	Random Forest (RF) classifier yields maximum accuracy, recall and AUC values. The hyperparameter tuning and Boruta algorithm added significance to the SVM and RF classification, thereby resulting in a F-score of 91% and 92% respectively	Logistic Regression-w/ imputation (accuracy = 78.95, recall = 70.00,AUC = 79.16), Logistic Regression-w/ dropna (accuracy = 75.00, recall = 70.00, AUC = 70.00), SVM (accuracy = 81.58, recall = 70.00, AUC = 82.22), Decision Tree (accuracy = 81.58, recall = 65.00, AUC = 82.50) Random Forest (accuracy = 84.21, recall = 80.00, AUC = 84.44), AdaBoost (accuracy = 84.21, recall = 65.00, AUC = 84.50)
64	Toshkhujaev et al. [78]	2020	To classify Alzheimer's disease using T1-weighted structural MRI	National Research Center for Dementia homepage, Alzheimer’s Disease Neuroimaging Initiative (ADNI), Alzheimer’s Disease Repository Without Borders, National Alzheimer’s Coordinating Center (701 paricipants)	Neurodegenrative Disease	Principal component analysis, support vector machine radial basis function classifier	A novel method for automatic classification, Alzheimer’s Disease from mild cognitive impairment and an health control was developed with more than 80% accuray for every dataset considered in the study	NA
65	Li and Yang [79]	2021	To build machine learning-based MRI data classifiers to predict Alzheimer's disease and infer the brain regions that contribute to disease development and progression and systematically compared the three distinct classifier	T1-weighted MR images from Alzheimers Disease Neuroimaging Initiative (ADNI) (560 participants)	Neurodegenrative Disease	Support Vector Machine, 3D Very Deep Convolutional Network (VGGNet) and 3D Deep Residual Network (ResNet) to improve the performance of classifiers—transfer learning strategy	The comparisons suggested that the ResNet model provided the best classification performance as well as more accurate localization of disease-associated regions in the brain compared to the VGGNet and support vector machine approaches	SVM (accuracy = 0.90, sensitivity = 0.939, specificity = 0.851, AUC = 0.97), 3D-VGGNet (accuracy = 0.95, sensitivity = 0.914, specificity = 1, AUC = 0.994), 3D-ResNet (accuracy = 0.95, sensitivity = 0.943, specificity = 0.96, AUC = 0.995)
66	Romero-Rosales et al. [80]	2020	The main objective of this research isto improve classification accuracy and extend the set of possible genetic risk factors for Alzheimer's disease	National Institute on Aging—Late-Onset Alzheimer’s Disease Family Study: Genome-Wide Association Study for Susceptibility Loci (phs000168.v2.p2) NCBI & Genotypes and Phenotypes database (dbGaP) (5,220 individuals)	Neurodegenrative Disease	Bootstrapping Stage-Wise Model Selection (BSWiMS), LASSO, GALGO	The addition ofmarkers from an initial model plus the markers ofthe model fitted tomisclassified samples improves the area under the receiving operative curve by around 5%, reaching ~ 0.84, which ishighly competitive using only genetic information	BSWiMS (accuracy = 0.686, sensitivity = 0.626, specificity = 0.734, AUC = 0.680), GALGO (accuracy = 0.720, sensitivity = 0.616, specificity = 0.800, AUC = 0.708), LASSO (accuracy = 0.766, sensitivity = 0.663, specificity = 0.825, AUC = 0.744)
67	Wang et al. [81]	2022	To identify risk factors for hospitalization outcomes that could be mitigated early to improve outcomes and impact overall quality of life	Hospital record (8407 patients)	Hospitalization outcome among dementia patients	Ensemble tree based model, logistic regression, decision tree, random forest, multilayer perceptron neural network	Top identified hospitalization outcome risk factors, mostly from medical history, include encephalopathy, number of medical problems at admission, pressure ulcers, urinary tract infections, falls, admission source, age, race, anemia, etc., with several overlaps in multi-dementia groups	AUC-ROC for tenfold cross validation is reported for models
68	Tsang et al. [82]	2020	To build a novel ML approach to predict hospitalization of dementia patients and to identify individual features	Electronic health records (59,298 patients)	Hospitalization outcome among dementia patients	Deep neural networks—entropy regularization with ensemble deep neural networks (ECNN), Random Forest	The discovery and heuristic evidence of correlation provide evidence for further clinical study of said medical events as potential novel indicators. There also remains great potential for adaption of ECNN within other medical big data domains as a data mining tool for novel risk factor identification	ECNN (TPR = 0.746, TNR = 0.7662, PPV = 0.766, NPV = 0.744, accuracy = 0.755), RF (TPR = 0.746, TNR = 0.714, PPV = 0.710, NPV = 0.750, accuracy = 0.729)
69	Revathi et al. [83]	2022	(i) Predicting people with possibilities of Alzheimer in their late life by doing careful analysis on various risk factors associated with Alzheimer’s. (ii) Conducting a neuropsychological test called Cognitive Ability Test (CAT) to assess the cognitive decline of a person	Clinical data (2361 patients)	Neurodegenrative Disease	Support vector machine, random forest, multinomial logistic regression	The study classified the risk factor using the operational definitions: “No Alzheimer’s,” “Uncertain Alzheimer’s,” and “Definite Alzheimer’s”. SVM of stage 1 classifier predicts with an accuracy of 0.86 and Random Forest with an accuracy of 0.71. Multinomial Logistic algorithm of stage 2 classifier accuracy is 0.89. 'e proposed work enables early prediction of a person at risk of Alzheimer’s Disease	Accuracy, sensitivity, specificity were reported for the tests and the models
70	Cooray et al. [84]	2021	1) To investigate the possibility of using ML to identify the most important predictors of tooth loss 2) to predict the incidence of tooth loss 3) to understand the behaviour of those predictors	19,407 older adult sfrom Japan Gerentological Evaluation Study (JAGES	Oral Diseases	Feature selection—Boruta algorithm ML models- XGBoost classification algorithm, Random forest classification model	XGBoost outperformed Random forest. Prediction of tooth loss was mainly influenced by older age, baseline oral health (having 10–19 teeth, wearing dentures), lower household income and manual occupations	XBoost (accuracy = 73.2%, F1 score = 21.4%, ROCAUC = 53.0%), RF (accuracy = 71.6%, F1 score = 25.3%, ROCAUC = 55.0%)

### Biological and brain age prediction

Biological age refers to an individual's age as determined by their physical and physiological health, as opposed to their chronological age brain age on the other hand, is a measurement of the age of an individual's brain based on its physical condition and function. The concept of biological age and brain age is useful in understanding an individual's overall health and risk of age-related diseases. By using neural networks and supervised ML algorithms on clinical datasets, biological age prediction [15] and brain age prediction [12] were conducted by two different studies. Ageing-related problems [13], measurement of healthy ageing [17], and the association between the health status of older adults and environmental and social factors [18–20] were studied extensively.

### Neurodegenerative disorders

There are many studies focused on neurodegenerative disorder, which includes Alzheimer’s disease, Parkinson’s disease, Dementia, and any type of cognitive disorders. Different types of deep neural networks were applied and compared with Cox proportional hazard models to predict the neurodegenerative disorders using population-based datasets [66], and another comparative analysis was done to understand the association between ageing process and these diseases [58]. Among all kinds of neurodegenerative diseases, Alzheimer’s disease was focused a lot. Some studies used biomarker data for example white matter hyperintensities [62], and particulate matter [70] to explore their role in causing Alzheimer’s disease. On the other hand, interestingly many studies have used social media (Twitter) data, to capture the sentiment of a large number of populations regarding the stigma of the disease [67]. Insurance claim data [57] is also used for the study. Most of the studies used regularised regression models, logistic regression, and deep learning models for capturing the risk. Among neurodegenerative disorders, another part of the disease is dementia [54, 61] and cognitive dysfunctions. Machine learning was used to diagnose and predict cognitive dysfunction mostly using population-based data [55, 56, 60, 72, 75], mostly using regression models from supervised ML, another type of studies have used biomarker variables [65], digital device features [59, 69], and hospital records [68] to analyse the risk factors of cognitive dysfunction. Similarly, for dementia, most of the studies used population-based surveys [63, 71, 73, 74] and clinical datasets [64] using classification and deep learning methods of ML. Mostly, logistic regression and random forest regression performed better than the other models applied.

### Non-communicable diseases

Among NCDs, diabetes, hypertension, chronic kidney diseases, cancer, and cardiovascular diseases were explored. Diabetes mellitus is very common among the older population and machine learning has been extensively applied for the detection, prediction, and identification of risk factors of the disease. Studies have developed predictive models [34, 35] based on supervised ML (logistic regression, XG Boost, decision tree, etc.) and some studies identified associated risk factors [32, 33] using clustering algorithms (like principal component analysis), logistic regression classifiers, and other supervised ML algorithms. Predicative models for hypertension were developed by using population-based datasets [39], with the association of risk factors like high waist circumference [38], cognitive impairment [37], and sleep & pulmonary measures [36] discussed. LASSO (Least Absolute Shrinkage and Selection Operator) and Ridge regression were popularly used for finding the association of the risk factors and feature section for model building. Chronic kidney disease detection [42], predicting stone-free status [40], and identifying distinct types of kidney transplants [41] were the focus areas. Patient records from the hospital were the only source for this kind of study. To detect chronic kidney disease, image processing, and deep learning algorithms were used, for predicting stone-free status, supervised ML algorithms (like logistic regression, random forest, and XG Boost regressor) were used, and lastly for identifying the kidney transplants clustering approach was used on the organ sharing data of patients. We found two studies based on colorectal cancer [22] which evaluated its risk using a random forest model on primary care health records. Another study predicted the quality of colorectal cancer surgery [24] with the 30-day mortality data from a hospital record and applied supervised ML algorithms. Many studies applied cross-sectional health survey data [25, 26, 28] and administrative databases [27, 29, 31] for predicting the risk of cardiovascular diseases. These studies used deep learning algorithms, cluster analysis, and ensemble ML to identify risk factors.

### Mental health conditions

Except physical health, mental health has equal importance for the overall body condition of older adults. Many types of mental health-related issues among the older population, for example, predicting depression from smartphone data using supervised ML models [46], identifying patients with depressive symptoms using random forest decision models on primary care visits [44], suicide prediction model [43], analysing the effect of environmental factors on mental health [45, 49], quantifying the psychotherapy content and its effect [47], and studying loneliness using social media data Twitter [48] and sentiment analysis were addressed by different studies. Most of the studies applied logistic regression, random forest, and deep learning models. The studies identifying environmental factors have used image data of surroundings along with neural networks and deep learning algorithms.

Furthermore, other diseases like oral health-related diseases, bone diseases, and multimorbidity were also covered. From our advanced search, we only found one study in the field of oral health, which predicted tooth loss among older adults using a population-based dataset and supervised ML classification algorithms [84]. One study on osteoporosis [21], which used CT (Computed Tomography) scan image data to develop various supervised ML models to develop prediction models was also found. Classification and prediction models for multimorbidity were developed using deep learning models, by comparing them with other ML algorithms [50]. For developing a score for an overall health condition, a set of multimorbidity was taken into account and analyzed with the help of logistic regression, and a gradient-boosted tree classifier [53].

### Type of methods and datasets used in the included studies

We've provided an overview of model performance metrics in the final column of Table 3. These metrics include the Area Under the Curve—Receiver Operating Characteristic (AUC-ROC), accuracy, specificity, sensitivity, precision, and the F1 score. Based on these performance scores, the Random Forest algorithm demonstrated the highest performance, with Extreme Gradient Boosting and Neural Networks following as the second and third-best performers, respectively. To clarify, the AUC-ROC measures the ability of a model to distinguish between different classes, accuracy represents the proportion of correctly classified instances, specificity measures the ability to correctly identify negative cases, sensitivity assesses the ability to correctly identify positive cases, precision quantifies the accuracy of positive predictions, and the F1 score combines precision and sensitivity to provide a balanced evaluation of the model's performance.

**Fig. 3:**
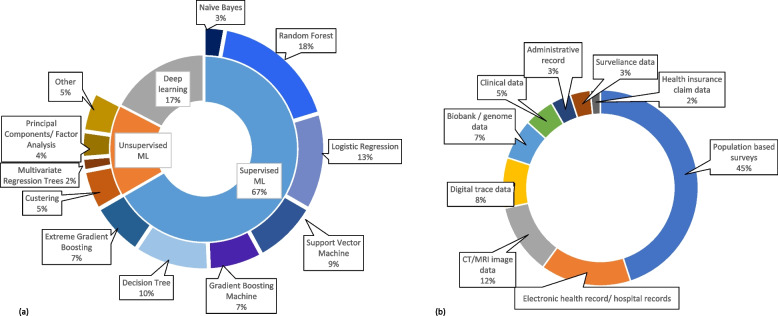
**a** Different machine learning methods used for geriatric diseases*. **b** Type of dataset used in the included studies * The total number of papers here is more than 70 because many papers have used more than one algorithm and are counted more than one time to make this chart

Figure 3 (a) & (b) shows the application of different types of ML methods and types of datasets used respectively. 67% of the papers included in our study have used supervised ML models, in which Random Forest, Logistic Regression, and the Decision Tree were the top three mostly used algorithms. After supervised ML, deep learning was the second most used algorithm, and lastly unsupervised ML. In unsupervised ML, the clustering technique was used often. Fig. 3 (b) shows the different types of datasets used. Nearly half of the studies (45%) used population-based surveys, while electronic health records and hospital-based data were the second highest used dataset. In the population-based datasets, two data sources English Longitudinal Study of Ageing (ELSA) and China Health and Retirement Longitudinal Study (CHARLS) were used more often. Then MRI/ CT images were the second highest used dataset. Many kinds of digitally traced data were also used, which includes internet databases like Kaggle, mobile application-based datasets, and data retrieved from social media APIs (Application Programming Interface) like Twitter.

The application of ML methods can be explained in three parts that is classification, risk prediction, and disease detection & diagnosis. The highest number of studies have predicted the risk factors or have built risk prediction models for different diseases. Under risk prediction, health survey datasets from population-based cross-sectional studies or biobank studies have been used to build the models. For the same, survival models like the Cox proportional hazard ratio model penalized regressions and supervised ML (decision tree, random forest, support vector machine, and logistic regression) were mostly used. Another type of data is nationally represented administrative population-based datasets, which have mostly focused on the sociodemographic and behavioral risk factors of the diseases. These studies have used supervised ML and deep learning methods to get the desired result. Except for these, some studies used hospital records, primary care visit records, and insurance data. The second category is disease detection and classification. Disease detection refers to the identification of the presence of a disease or condition usually through screening and testing. While, on the other hand, disease diagnosis is the process of identifying the specific disease or condition that is causing a person’s symptoms [85]. Generally, population-based datasets, biomarker data, and image-based data such as MRI, and retinal image data were used to detect or diagnose the disease in the studies. For image-based data, hierarchical clustering is used to detect the disease. In the case of other datasets, a mix of deep learning and supervised machine learning was used. The third category is disease classification. In the context of machine learning, disease classification refers to the process of developing algorithms or models that can automatically classify or categorize diseases based on labeled data separating diseases and not diseases or the severity of the illness. Ensemble ML algorithms, deep neural networks, random forests, and decision trees were the methods used for the classification of diseases using the health survey datasets containing phenotype data and national health survey datasets [86].

## Discussion

The ML methods have been largely used to study different types of illness and in different kinds of datasets. The use of machine learning in the development of new treatments and interventions leads to the development of new drugs that can slow down the disease progression process [87]. The exponential increase in the use of machine learning in geriatric care is evidenced by the growing number of research studies in this field. There has been a significant number of published studies identified in the last decade and it’s growing day by day.

Machine learning is a relatively new technology in the field of geriatrics, and it has the potential to revolutionize the way we diagnose, treat, and manage geriatric diseases. In comparison to traditional methods, machine learning has several advantages that make it a promising tool for geriatric care. One of the most important advantages of ML is its ability to analyse a large amount of unstructured data like image data CT scans, MRI, tumor images, etc. It can find patterns and detect those morbidities quickly and more accurately [88–90]. While traditional methods rely on manual data analysis which is time-consuming and prone to human error, ML algorithms with their ability to learn and adapt, can be trained on large datasets and improve their accuracy over time [91]. In contrast, traditional methods rely on fixed rules and protocols that may not be able to adapt to the changing needs of geriatric patients. Machine learning algorithms can analyse patient data to identify those at high risk of developing a disease. This early identification can enable clinicians to intervene early and provide appropriate treatment to prevent or slow down the progression of the disease [92]. A paper by Ali et al. systematically reviewed 180 research articles according to the application of artificial intelligence in healthcare benefits, challenges, methodologies, and functionalities, which concluded that this novel method continues to outperform humans in terms of accuracy, efficiency, and fast execution of clinical processes [93]. Another systematic literature review by Battineni et al. suggested that in real-time clinical practice, there is no universally accepted approach for determining the optimal method, as each machine learning technique comes with its own set of strengths and limitations, however, Support Vector Machines (SVM) and Logistic Regression (LR) are two common machine learning methods that are used in most of the studies [94]. In another review article, the majority of the examined studies emphasized that the use of only machine learning methods or combining it with other intelligent techniques is popularly used to prevent emergencies [95]. This approach holds a significant promise for uncovering substantial patterns in both structured and unstructured datasets. The widespread adoption of these techniques generates curiosity about their global evolution and which countries utilize them most extensively. According to Tran et al., the trend of usage of machine learning and artificial intelligence in research is highest in the United States, followed by China and Italy [96]. Previous systematic literature studies have highlighted mostly clinical aspects of geriatrics and have only focused on chronic diseases [8, 10] and some have focused only on mental health disorders [9] or frailty [11]. The current study covers the total breadth of ageing from diseases to mental health problems and also the successful ageing, brain age, and biological age prediction. The study has also included literature which have used population-based surveys to build a successful ageing index by exploring new methods and datasets. Many kinds of digitally traced data [30, 46–48, 67, 69] are used in studies that have a future scope of application for improving geriatric research.

However, it is important to note that machine learning is not without its limitations. ML algorithms require large amounts of high-quality data to be effective, and there may be issues with data quality or bias that can impact the accuracy of the algorithms. Additionally, machine learning algorithms are not always transparent in their decision-making [97], which can make it difficult for clinicians to understand how the algorithms arrived at a particular diagnosis or treatment recommendation. So, in most cases, it may be suitable to use both the conventional methods and the ML methods side by side to get better results [98]. To summarize, machine learning offers several benefits in geriatric care such as its ability to rapidly and accurately analyse large amounts of data, learn and improve over time, and enhance the precision of diagnoses and treatment recommendations. However, it is crucial to acknowledge the potential limitations of machine learning and take necessary measures to ensure that the algorithms are fair, transparent, and validated before their implementation in clinical settings. The future scope of ML in geriatrics is vast and promising. With the aging population on the rise globally, there is a growing need for innovative technologies that can enhance the quality of care for the elderly. However, it is crucial to continue research and development to ensure that the algorithms are fair, transparent, and validated before their widespread implementation in clinical practice.

## Conclusion

The current review found a wide variety of research papers analyzing different diseases using various machine learning algorithms in different kinds of datasets. Disease diagnostic criteria, risk prediction models, and factors were also highlighted and the application of machine learning in the field of geriatrics and care is well explored, but still, there is scope for future development. There is a need to validate that constructed machine learning models in large-scale datasets generalize the results across all age groups, gender, ethnicity, and other crucial factors. Also, there is a huge scope in using internet-based data and digital datasets from personalized applications in digital devices like smartphones and wearable technologies to provide customized patient-centric care for older populations.

### Supplementary Information

**Additional file 1: Table S1.** Research papers included in the review. **Table S2.** Risk of bias assessed by Joanna Briggs Institute (JBI) Critical Appraisal Tools.

## Data Availability

All data generated or analyzed during this study are included in this published article [and Table- S1 of the supplementary information files].
