# Supplementary material for: Application of machine learning in measurement of ageing and geriatric diseases: a systematic review
Source: BMC Geriatr. 2023 Dec 12;23:841. doi: 10.1186/s12877-023-04477-x (PMC10717316; doi:10.1186/s12877-023-04477-x)
Supplement: Supplementary file 1 — Additional file 1: Table S1. Research papers included in the review. Table S2. Risk of bias assessed by Joanna Briggs Institute (JBI) Critical Appraisal Tools. [file 12877_2023_4477_MOESM1_ESM.docx]

**Table S1: Research papers included in the review**

|  | **Paper title** | **Author** | **Year published** | **DOI** |
| --- | --- | --- | --- | --- |
| 1 | A comparison of machine learning methods for survival analysis of high-dimensional clinical data for dementia prediction | Spooner et al. | 2020 | 10.1038/s41598-020-77220-w |
| 2 | A composite sleep and pulmonary phenotype predicting hypertension | Li et al. | 2021 | 10.1016/j.ebiom.2021.103433 |
| 3 | A deep learning algorithm to detect chronic kidney disease from retinal photographs in community-based populations | Sabanayagam et al. | 2020 | 10.1016/S2589-7500(20)30063-7 |
| 4 | A Machine Learning Approach for Early Diagnosis of Cognitive Impairment Using Population-Based Data | Tan et al. | 2023 | 10.3233/JAD-220776 |
| 5 | A retrospective cohort study on the use of machine learning to predict stone-free status following percutaneous nephrolithotomy: An experience from Saudi Arabia | Alghafees et al. | 2022 | 10.1016/j.amsu.2022.104957 |
| 6 | A Risk Prediction Model Based on Machine Learning for Cognitive Impairment Among Chinese Community-Dwelling Elderly People With Normal Cognition: Development and Validation Study | Hu et al. | 2021 | 10.2196/20298 |
| 7 | A risk prediction model based on machine learning for early cognitive impairment in hypertension: Development and validation study | Zhong et al. | 2023 | 10.3389/fpubh.2023.1143019 |
| 8 | Advanced analytical methodologies for measuring healthy ageing and its determinants, using factor analysis and machine learning techniques: the ATHLOS project | Caballero et al. | 2017 | 10.1038/srep43955 |
| 9 | Alzheimer-type dementia prediction by sparse logistic regression using claim data | Fukunishi et al. | 2020 | 10.1016/j.cmpb.2020.105582 |
| 10 | Application of ensemble machine learning algorithms on lifestyle factors and wearables for cardiovascular risk prediction | Huang et al. | 2022 | 10.1038/s41598-021-04649-y |
| 11 | Application of machine learning model to predict osteoporosis based on abdominal computed tomography images of the psoas muscle: a retrospective study | Huang et al. | 2022 | 10.1186/s12877-022-03502-9 |
| 12 | Classification and Prediction on the Effects of Nutritional Intake on Overweight/Obesity, Dyslipidemia, Hypertension and Type 2 Diabetes Mellitus Using Deep Learning Model: 4-7th Korea National Health and Nutrition Examination Survey | Kim et al. | 2021 | 10.3390/ijerph18115597 |
| 13 | Comparative Analysis of Multiple Neurodegenerative Diseases Based on Advanced Epigenetic Aging Brain | Shi et al. | 2021 | 10.3389/fgene.2021.657636 |
| 14 | Comparison of Biological Age Prediction Models Using Clinical Biomarkers Commonly Measured in Clinical Practice Settings: AI Techniques Vs. Traditional Statistical Methods, | Kim and Kim | 2021 | 10.3389/frans.2021.709589 |
| 15 | Computer-Aided Diagnosis of Alzheimer's Disease through Weak Supervision Deep Learning Framework with Attention Mechanism | Liang and Gu | 2020 | 10.3390/s21010220 |
| 16 | Data-driven decision making for the screening of cognitive impairment in primary care: a machine learning approach using data from the ELSA-Brasil study | Szlejf et al. | 2023 | 10.1590/1414-431X2023e12475 |
| 17 | Decoding expectation and surprise in dementia: the paradigm of music | Benhamou et al. | 2021 | 10.1093/braincomms/fcab173 |
| 18 | Developing the Total Health Profile, a Generalizable Unified Set of Multimorbidity Risk Scores Derived From Machine Learning for Broad Patient Populations: Retrospective Cohort Study | Mahajan et al. | 2021 | 10.2196/32900 |
| 19 | Development of a Suicide Prediction Model for the Elderly Using Health Screening Data | Cho et al. | 2021 | 10.3390/ijerph181910150 |
| 20 | Development of Nonlaboratory-Based Risk Prediction Models for Cardiovascular Diseases Using Conventional and Machine Learning Approaches | Sajid et al. | 2021 | 10.3390/ijerph182312586 |
| 21 | Evaluation of a prediction model for colorectal cancer: retrospective analysis of 2.5 million patient records | Birks et al. | 2017 | 10.1002/cam4.1183 |
| 22 | Extracting and summarizing white matter hyperintensities using supervised segmentation methods in Alzheimer's disease risk and aging studies | Ithapu et al. | 2014 | 10.1002/hbm.22472 |
| 23 | Health status prediction for the elderly based on machine learning | Qin et al. | 2020 | 10.1016/j.archger.2020.104121 |
| 24 | High waist circumference is a risk factor of new-onset hypertension: Evidence from the China Health and Retirement Longitudinal Study | Sun et al. | 2022 | 10.1111/jch.14446 |
| 25 | Hyperglycemia screening based on survey data: an international instrument based on WHO STEPs dataset | Moradifar et al. | 2022 | 10.1186/s12902-022-01222-0 |
| 26 | Identification of High Likelihood of Dementia in Population-Based Surveys using Unsupervised Clustering: a Longitudinal Analysis | Gharbi-Meliani et al. | 2023 | 10.1101/2023.02.17.23286078 |
| 27 | Identification of Patients in Need of Advanced Care for Depression Using Data Extracted From a Statewide Health Information Exchange: A Machine Learning Approach | Kasthurirathne et al. | 2019 | 10.2196/13809 |
| 28 | Identifying undetected dementia in UK primary care patients: a retrospective case-control study comparing machine-learning and standard epidemiological approaches | Ford et al. | 2019 | 10.1186/s12911-019-0991-9 |
| 29 | Importance of socioeconomic factors in predicting tooth loss among older adults in Japan: Evidence from a machine learning analysis | Cooray et al. | 2021 | 10.1016/j.socscimed.2021.114486 |
| 30 | Improved perfusion pattern score association with type 2 diabetes severity using machine learning pipeline: Pilot study | Chen et al. | 2019 | 10.1002/jmri.26256 |
| 31 | Investigating Predictors of Cognitive Decline Using Machine Learning | Casanova et al. | 2020 | 10.1093/geronb/gby054 |
| 32 | Investigating the Association between Streetscapes and Mental Health in Zhanjiang, China: Using Baidu Street View Images and Deep Learning | Zhang et al. | 2022 | 10.3390/ijerph192416634 |
| 33 | Machine learning for predicting neurodegenerative diseases in the general older population: a cohort study | Aguayo et al. | 2023 | 10.1186/s12874-023-01837-4 |
| 34 | Machine Learning, Sentiment Analysis, and Tweets: An Examination of Alzheimer’s Disease Stigma on Twitter | Oscar et al. | 2017 | 10.1093/geronb/gbx014 |
| 35 | Machine Learning-Derived Echocardiographic Phenotypes Predict Heart Failure Incidence in Asymptomatic Individuals | Kobayashi et al. | 2022 | 10.1016/j.jcmg.2021.07.004 |
| 36 | Measuring neuropsychiatric symptoms in patients with early cognitive decline using speech analysis | König et al. | 2021 | 10.1192/j.eurpsy.2021.2236 |
| 37 | Modeling Users' Cognitive Performance Using Digital Pen Features | Prange and Sonntag et al. | 2022 | 10.3389/frai.2022.787179 |
| 38 | Particulate matter and episodic memory decline mediated by early neuroanatomic biomarkers of Alzheimer's disease | Younan et al. | 2020 | 10.1093/brain/awz348 |
| 39 | Predicting cardiovascular risk from national administrative databases using a combined survival analysis and deep learning approach | Barbieri et al. | 2022 | 10.1093/ije/dyab258 |
| 40 | Predicting Cognitive Impairment and Dementia: A Machine Learning Approach | Aschwanden et al. | 2020 | 10.3233/JAD-190967 |
| 41 | Predicting Depression From Smartphone Behavioral Markers Using Machine Learning Methods, Hyperparameter Optimization, and Feature Importance Analysis: Exploratory Study | Asare et al. | 2021 | 10.2196/26540 |
| 42 | Predicting hypertension using machine learning: Findings from Qatar Biobank Study | Alkaabi et al. | 2020 | 10.1371/journal.pone.0240370 |
| 43 | Prediction of Decline in Global Cognitive Function Using Machine Learning with Feature Ranking of Gait and Physical Fitness Outcomes in Older Adults | Noh et al. | 2021 | 10.3390/ijerph182111347 |
| 44 | Prediction of type 2 diabetes mellitus using hematological factors based on machine learning approaches: a cohort study analysis | Mansoori et al. | 2023 | 10.1038/s41598-022-27340-2 |
| 45 | Predictive models for diabetes mellitus using machine learning techniques | Lai et al. | 2019 | 10.1186/s12902-019-0436-6 |
| 46 | Predictors of 30-Day Mortality Among Dutch Patients Undergoing Colorectal Cancer Surgery, 2011-2016 | Bosch et al. | 2021 | 10.1001/jamanetworkopen.2021.7737 |
| 47 | Predictors of Dementia in the Oldest Old: A Novel Machine Learning Approach | Jia et al. | 2020 | 10.1097/WAD.0000000000000400 |
| 48 | Protocol for a conversation-based analysis study: PREVENT-ED investigates dialogue features that may help predict dementia onset in later life | Garcia et al. | 2019 | 10.1136/bmjopen-2018-026254 |
| 49 | Quantifying the Association Between Psychotherapy Content and Clinical Outcomes Using Deep Learning | Ewbank et al. | 2020 | 10.1001/jamapsychiatry.2019.2664 |
| 50 | Sociodemographic Indicators of Health Status Using a Machine Learning Approach and Data from the English Longitudinal Study of Aging (ELSA) | Engchuan et al. | 2019 | 10.12659/MSM.913283 |
| 51 | Studying expressions of loneliness in individuals using twitter: an observational study | Guntuku et al. | 2019 | 10.1136/bmjopen-2019-030355 |
| 52 | The ideal neighbourhoods of successful ageing: A machine learning approach | Wong et al. | 2021 | 10.1016/j.healthplace.2021.102704 |
| 53 | Use of Machine Learning Consensus Clustering to Identify Distinct Subtypes of Kidney Transplant Recipients With DGF and Associated Outcomes | Jadlowiec et al. | 2022 | 10.3389/ti.2022.10810 |
| 54 | Using deep learning to examine street view green and blue spaces and their associations with geriatric depression in Beijing, China | Helbich et al. | 2019 | 10.1016/j.envint.2019.02.013 |
| 55 | Using machine learning models to improve stroke risk level classification methods of China national stroke screening | Li et al. | 2019 | 10.1186/s12911-019-0998-2 |
| 56 | Using Machine Learning to Predict Cognitive Impairment Among Middle-Aged and Older Chinese: A Longitudinal Study | Liu et al. | 2023 | 10.3389/ijph.2023.1605322 |
| 57 | Validation of a deep-learning-based retinal biomarker (Reti-CVD) in the prediction of cardiovascular disease: data from UK Biobank | Tseng et al. | 2023 | 10.1186/s12916-022-02684-8 |
| 58 | A new strategy for the early detection of alzheimer disease stages using multifractal geometry analysis based on K‑Nearest Neighbor algorithm | Elgammal et al. | 2022 | 10.1038/s41598-022-26958-6 |
| 59 | Alzheimer Disease Detection Empowered with Transfer Learning | Ghazal et al. | 2021 | 10.32604/cmc.2022.020866 |
| 60 | Alzheimer’s Dementia: Diagnosis and Prognosis using Neuro-Imaging Analysis | Sountharrajan et al. | 2022 | 10.47750/pnr.2022.13.04.006 |
| 61 | Classification of Alzheimer’s Disease and Mild Cognitive Impairment Based on Cortical and Subcortical Features from MRI T1 Brain Images Utilizing Four Different Types of Datasets | Toshkhujaev et al. | 2020 | 10.1155/2020/3743171 |
| 62 | Comparison of machine learning approaches for enhancing Alzheimer’s disease classification | Li & Yang | 2021 | 10.7717/peerj.10549 |
| 63 | Improving predictive models for Alzheimer’s disease using GWAS data by incorporating misclassified samples modeling | Romero-Rosales et al. | 2020 | 10.1371/journal.pone.0232103 |
| 64 | Risk factors and machine learning model for predicting hospitalization outcomes in geriatric patients with dementia | Wang et al. | 2022 | 10.1002/trc2.12351 |
| 65 | Modeling Large Sparse Data for Feature Selection: Hospital Admission Predictions of the Dementia Patients Using Primary Care Electronic Health Records | Tsang et al. | 2020 | 10.1109/JTEHM.2020.3040236 |
| 66 | Early Detection of Cognitive Decline Using Machine Learning Algorithm and Cognitive Ability Test | Revathi et al. | 2022 | 10.1155/2022/4190023 |
| 67 | Neuroimaging-derived brain age is associated with life satisfaction in cognitively unimpaired elderly: A community-based study | Sone et al. | 2022 | 10.1038/s41398-022-01793-5 |
| 68 | Cardiovascular risk prediction in healthy older people | Neumann et al. | 2021 | 10.1007/s11357-021-00486-z |
| 69 | Gait Speed and Survival of Older Surgical Patient with Cancer: Prediction after Machine Learning | Sasani et al. | 2019 | 10.1016/j.jgo.2018.06.012 |
| 70 | Predicting the Anxiety of Patients with Alzheimer’s Dementia using Boosting Algorithm and Data-Level Approach | Byeon | 2021 | 10.14569/IJACSA.2021.0120313 |
|  |  |  |  |  |
|  |  |  |  |  |

A total of 72 papers were chosen for inclusion in the review after a thorough search and selection process utilizing the PubMed and Scopus search engine.

**Table S2: Risk of bias assessed by Joanna Briggs Institute (JBI) Critical Appraisal Tools**

| **Author** | **Questions on JBI critical appraisal checklist** | | | | | | | | **% of yes** | **Risk** |
| --- | --- | --- | --- | --- | --- | --- | --- | --- | --- | --- |
|  | **Q1** | **Q2** | **Q3** | **Q4** | **Q5** | **Q6** | **Q7** | **Q8** |  |  |
| Spooner et al. | 1 | 1 | 1 | 1 | 0 | 0 | 1 | 1 | 75 | Low risk |
| Li et al. | 1 | 1 | 1 | 1 | 0 | 0 | U | 1 | 62.5 | Moderate risk |
| Sabanayagam et al. | 1 | 1 | 1 | 1 | 0 | 0 | 1 | 1 | 75 | Low risk |
| Tan et al. | 1 | 1 | 1 | 1 | 0 | 0 | 1 | 1 | 75 | Low risk |
| Alghafees et al. | 1 | 1 | 1 | 1 | 0 | 0 | 1 | 1 | 75 | Low risk |
| Hu et al. | 1 | 1 | 1 | 1 | 0 | 0 | 1 | 1 | 75 | Low risk |
| Zhong et al. | 1 | 1 | 1 | 1 | 0 | 0 | 1 | 1 | 75 | Low risk |
| Caballero et al. | 1 | 1 | 1 | 1 | 0 | 0 | 1 | 1 | 75 | Low risk |
| Fukunishi et al. | 1 | 1 | 1 | 1 | 0 | 0 | 1 | 1 | 75 | Low risk |
| Huang et al. | 1 | 1 | 1 | 1 | 1 | 1 | 1 | 1 | 100 | Low risk |
| Huang et al. | 1 | 1 | 1 | 1 | 0 | 0 | 1 | 1 | 75 | Low risk |
| Kim et al. | 1 | 1 | 1 | 1 | 0 | 0 | 1 | 1 | 75 | Low risk |
| Shi et al. | 1 | 1 | 1 | 1 | 0 | 0 | 1 | 1 | 75 | Low risk |
| Kim and Kim | 1 | 1 | 1 | 1 | 0 | 0 | 1 | 1 | 75 | Low risk |
| Liang and Gu | 1 | 1 | 1 | 1 | 0 | 0 | 1 | 1 | 75 | Low risk |
| Szlejf et al. | 1 | 1 | 1 | 1 | 0 | 0 | 1 | 1 | 75 | Low risk |
| Benhamou et al. | 1 | 1 | 1 | 1 | 1 | 1 | 1 | 1 | 100 | Low risk |
| Mahajan et al. | 1 | 1 | 1 | 1 | 0 | 0 | 1 | 1 | 75 | Low risk |
| Cho et al. | 1 | 1 | 1 | 1 | 0 | 0 | U | 1 | 62.5 | Moderate risk |
| Sajid et al. | 1 | 1 | 1 | 1 | 1 | 1 | 1 | 1 | 100 | Low risk |
| Birks et al. | 1 | 1 | 1 | 1 | 0 | 0 | 1 | 1 | 75 | Low risk |
| Ithapu et al. | 1 | 1 | 1 | 1 | 0 | 0 | 1 | 1 | 75 | Low risk |
| Qin et al. | 1 | 1 | 1 | 1 | 0 | 0 | 1 | 1 | 75 | Low risk |
| Sun et al. | 1 | 1 | 1 | 1 | 0 | 0 | 1 | 1 | 75 | Low risk |
| Moradifar et al. | 1 | 1 | 1 | 1 | 0 | 0 | 1 | 1 | 75 | Low risk |
| Gharbi-Meliani et al. | 1 | 1 | 1 | 1 | 0 | 0 | 1 | 1 | 75 | Low risk |
| Kasthurirathne et al. | 1 | 1 | 1 | 1 | 0 | 0 | U | 1 | 62.5 | Moderate risk |
| Ford et al. | 1 | 1 | 1 | 1 | 0 | 0 | 1 | 1 | 75 | Low risk |
| Cooray et al. | 1 | 1 | 1 | 1 | 0 | 0 | 1 | 1 | 75 | Low risk |
| Chen et al. | 1 | 1 | 1 | 1 | 0 | 0 | 1 | 1 | 75 | Low risk |
| Casanova et al. | 1 | 1 | 1 | 1 | 0 | 0 | 1 | 1 | 75 | Low risk |
| Zhang et al. | 1 | 1 | 1 | 1 | 0 | 0 | 1 | 1 | 75 | Low risk |
| Aguayo et al. | 1 | 1 | 1 | 1 | 0 | 0 | 1 | 1 | 75 | Low risk |
| Oscar et al. | 1 | 1 | 1 | 1 | 0 | 0 | 1 | 1 | 75 | Low risk |
| Kobayashi et al. | 1 | 1 | 1 | 1 | 0 | 0 | 1 | 1 | 75 | Low risk |
| König et al. | 1 | 1 | 1 | 1 | 0 | 0 | 1 | 1 | 75 | Low risk |
| Prange and Sonntag et al. | 1 | 1 | 1 | 1 | 0 | 0 | 1 | 1 | 75 | Low risk |
| Younan et al. | 1 | 1 | 1 | 1 | 1 | 1 | 1 | 1 | 100 | Low risk |
| Barbieri et al. | 1 | 1 | 1 | 1 | 0 | 0 | 1 | 1 | 75 | Low risk |
| Aschwanden et al. | 1 | 1 | 1 | 1 | 0 | 0 | 1 | 1 | 75 | Low risk |
| Asare et al. | 1 | 1 | 1 | 1 | 0 | 0 | 1 | 1 | 75 | Low risk |
| Alkaabi et al. | 1 | 1 | 1 | 1 | 0 | 0 | 1 | 1 | 75 | Low risk |
| Noh et al. | 1 | 1 | 1 | 1 | 0 | 0 | 1 | 1 | 75 | Low risk |
| Mansoori et al. | 1 | 1 | 1 | 1 | 0 | 0 | 1 | 1 | 75 | Low risk |
| Lai et al. | 1 | 1 | 1 | 1 | 0 | 0 | 1 | 1 | 75 | Low risk |
| Bosch et al. | 1 | 1 | 1 | 1 | 0 | 0 | 1 | 1 | 75 | Low risk |
| Jia et al. | 1 | 1 | 1 | 1 | 0 | 0 | 1 | 1 | 75 | Low risk |
| Garcia et al. | 1 | 1 | 1 | 1 | 1 | 0 | 1 | 1 | 87.5 | Low risk |
| Ewbank et al. | 1 | 1 | 1 | 1 | 0 | 0 | 1 | 1 | 75 | Low risk |
| Engchuan et al. | 1 | 1 | 1 | 1 | 1 | 0 | 1 | 1 | 87.5 | Low risk |
| Guntuku et al. | 1 | 1 | 1 | 1 | 0 | 0 | 1 | 1 | 75 | Low risk |
| Wong et al. | 1 | 1 | 1 | 1 | 0 | 0 | 1 | 1 | 75 | Low risk |
| Jadlowiec et al. | 1 | 1 | 1 | 1 | 0 | 0 | 1 | 1 | 75 | Low risk |
| Helbich et al. | 1 | 1 | 1 | 1 | 0 | 0 | 1 | 1 | 75 | Low risk |
| Li et al. | 1 | 1 | 1 | 1 | 0 | 0 | 1 | 1 | 75 | Low risk |
| Liu et al. | 1 | 1 | 1 | 1 | 0 | 0 | 1 | 1 | 75 | Low risk |
| Tseng et al. | 1 | 1 | 1 | 1 | 0 | 0 | 1 | 1 | 75 | Low risk |
| Elgammal et al. | 1 | 1 | 1 | 1 | 0 | 0 | 1 | 1 | 75 | Low risk |
| Ghazal et al. | 1 | 1 | 1 | 1 | 0 | 0 | 1 | 1 | 75 | Low risk |
| Sountharrajan et al. | 1 | 1 | 1 | 1 | 0 | 0 | 1 | 1 | 75 | Low risk |
| Toshkhujaev et al. | 1 | 1 | 1 | 1 | 0 | 0 | 1 | 1 | 75 | Low risk |
| Li & Yang | 1 | 1 | 1 | 1 | 0 | 0 | 1 | 1 | 75 | Low risk |
| Romero-Rosales et al. | 1 | 1 | 1 | 1 | 0 | 0 | 1 | 1 | 75 | Low risk |
| Wang et al. | 1 | 1 | 1 | 1 | 0 | 0 | 1 | 1 | 75 | Low risk |
| Tsang et al. | 1 | 1 | 1 | 1 | 0 | 0 | 1 | 1 | 75 | Low risk |
| Revathi et al. | 1 | 1 | 1 | 1 | 0 | 0 | 1 | 1 | 75 | Low risk |
| Sone et al. | 1 | 1 | 1 | 1 | 0 | 0 | 1 | 1 | 75 | Low risk |
| Neumann et al. | 1 | 1 | 1 | 1 | 0 | 0 | 1 | 1 | 75 | Low risk |
| Sasani et al. | 1 | 1 | 1 | 1 | 0 | 0 | 1 | 1 | 75 | Low risk |
| Byeon | 1 | 1 | 1 | 1 | 0 | 0 | 1 | 1 | 75 | Low risk |
|  |  |  |  |  |  |  |  |  |  |  |
|  |  |  |  |  |  |  |  |  |  |  |

Questions asked in the checklist: Q1. Were the criteria for inclusion in the sample clearly defined? Q2. Were the study subjects and the setting described in detail? Q3. Was the exposure measured in a valid and reliable way? Q4. Were objective, standard criteria used for measurement of the condition? Q5. Were confounding factors identified? Q6. Were strategies to deal with confounding factors stated? Q7. Were the outcomes measured in a valid and reliable way? Q8. Was appropriate statistical analysis used?

Abbreviations: 1 = Yes; 0 = No; U = Unclear

**Criteria used to rank the risk of bias**

i) ≤49% = high risk of Bias (0 Studies)

ii) 50% and 69% = Moderate risk of Bias (4 studies)

iii) Above 70% = low risk of Bias (68 studies)
